# Chemical Profile and Skin-Beneficial Activities of the Petal Extracts of *Paeonia tenuifolia* L. from Serbia

**DOI:** 10.3390/ph15121537

**Published:** 2022-12-11

**Authors:** Natalija Čutović, Tatjana Marković, Marina Kostić, Uroš Gašić, Željana Prijić, Xiuxia Ren, Milan Lukić, Branko Bugarski

**Affiliations:** 1 Institute for Medicinal Plants Research “Dr Josif Pančić”, Tadeuša Košćuška 1, 11000 Belgrade, Serbia; 2 Department of Plant Physiology, Institute for Biological Research “Siniša Stanković”—National Institute of Republic of Serbia, University of Belgrade, Bulevar Despota Stefana 142, 11000 Belgrade, Serbia; 3 Key Laboratory of Biology and Genetic Improvement of Horticultural Crops, Institute of Vegetables and Flowers, Chinese Academy of Agricultural Sciences, Ministry Agriculture and Rural Affairs, Beijing 100081, China; 4 Faculty of Technology and Metallurgy, University of Belgrade, Karnegijeva 4, 11000 Belgrade, Serbia

**Keywords:** polyphenols, antioxidant activity, antimicrobial activity, wound healing, herbaceous peony, Steppe peony

## Abstract

Without being aware of its chemical makeup, many ancient societies have used Steppe peony in their traditional medicine. Given that modern phytopreparation intended for use on human skin requires, above all, knowledge of its chemical composition, the goal of this study was to make a screening of the composition of aqueous and methanolic extracts of the petals of *P. tenuifolia* L. and to examine them for various skin-beneficial properties. The extracts were prepared by maceration, ultrasound-assisted, and microwave-assisted extraction procedures. The chemical profiling was conducted by the use of UHPLC-LTQ-OrbiTrap MS and UHPLC/MS, and spectrophotometric methods for the determination of total polyphenol and total flavonoid contents. The biological activities entailed antioxidant ABTS, DPPH, CUPRAC (Cupric Ion Reducing Antioxidant Capacity), and FRAP (Ferric Reducing Antioxidant Power) assays, antimicrobial (antibacterial and antifungal) and antibiofilm activities, cytotoxicity, wound healing potential, as well as the adhesion and invasion of *Staphylococcus lugdunensis*. The results showed that the petals are rich in phenolic acids and flavonoids, which are commonly associated with numerous biological activities. The aqueous extracts were more efficient in the majority of the bioactivity assays then the methanolic ones, whereas the optimal extraction method varied between the assays. This study is the first step towards the safe use of the aqueous extracts of *P. tenuifolia* petals for therapeutic skin treatments.

## 1. Introduction

Horticulturists, plant taxonomists, and herbalists have all paid close attention to peonies, since they are aware of their priceless medicinal and edible qualities in addition to their great ornamental and monetary value [1]. *Paeonia* L. is the only genus in the *Paeoniaceae* family, with 34 species native to the northern hemisphere [2]. The genus is divided into three sections. The section *Paeoniae* comprises 27 Eurasian herbaceous species, and five of them are wild growing in Serbia, namely *Paeonia officinalis* L. (European or female peony), *Paeonia banatica* Rochel (Pannonian peony), *Paeonia daurica* Andrews (Balkan or wild peony), *Paeonia peregrina* Mill. (Kosovo’s peony), and *Paeonia tenuifolia* (Steppe peony) [3,4]. Although the latter two are more spread across Serbia than the other three species, all of them are listed in the Red Book of the Flora of Serbia [5] and strictly protected by the Law on Nature Protection [6]. 

*P. tenuifolia* L. is a perennial herbaceous species also known as the fern leaf peony. It is native to Russia’s Caucasus Mountains and Ukraine’s Black Sea coast, and it has spread eastward to northwestern Kazakhstan and westward into Bulgaria, Romania, and Serbia. In Serbia, it spontaneously grows at a few localities, including, most abundantly, in Deliblato sands (in Banat, province of Vojvodina), but it can also be found in Central Serbia (in the vicinity of Dimitrovgrad, close to Vidlič mountain, and Sokobanja, in the municipality of Knjaževac, in the Zaječar District). It is a perennial herbaceous plant that grows up to 50 cm tall in forests and steppes. It has characteristic red solitary and terminal flowers composed of 5–8 obovate petals, which commonly bloom in May during a very short period of time lasting only 7–15 days [2,7]. 

People have used plant-based remedies to treat or prevent ailments for thousands of years, thanks to biologically active compounds deposited in their different organs (leaves, bark, fruits, roots, etc.). Among the organs, from time immemorial, the flowers have attracted a huge research interest, not only as a source of new biologically active constituents beneficial for their medical and cosmetic purposes, but also for culinary uses, to improve the nutritional, sensorial, and aesthetic qualities of food [8,9,10]. Various edible flowers, including some herbaceous peony species, contain a number of phytochemicals, including sugars, acids, proteins, minerals, flavonoids, polyphenols, anthocyanins, carotenoids, and fibers [11,12], which, apart from some biological properties, may also possess antioxidant capacity [13] and are ultimately beneficial to consumers’ health. 

Recent reviews on the genus *Paeonia* identified polyphenols as their main constituents and summarized several biological activities (antioxidant, anti-inflammatory, anticancer, and cardioprotective) of the *Paeonia* species [14,15]. There are numerous polyphenols of plant origin, but most likely, only a small portion of them are important for human health. Among them, flavonoids attract particular research interests.

In response to the growing concerns about the presence of multidrug-resistant bacteria and the subsequent decline in the efficacy of manufactured medications, interest in plants and their bioactive metabolites has increased [16,17]. Plants that are rich in bioactive molecules are widely used in the traditional medicine of various nations [18]. In recent times, many of them have been scientifically confirmed, and many new roles have also been discovered, including oxidative stress reduction capacity [19], antifungal and antibacterial efficacy [11,20], as well as wound healing capability [21]. These findings draw on innovative and in-depth studies of plant-based medications. If such medications are intended for use on human skin, in addition to the above-mentioned assays, antibiofilm activity, a cytotoxicity assay, and adhesion and invasion of *S. lugdunensis*, should also be included. The antioxidant activity suggests that the extracts could be used to alleviate the effects of oxidative stress caused by UV radiation exposure, which can lead to premature skin aging and a variety of skin diseases [22]. The antimicrobial and antibiofilm activities demonstrate the extract’s ability to inhibit the growth of microorganisms already present on the skin surface, as their abundance in individuals with compromised immune systems or damaged skin may cause serious health issues [23]. The wound-healing assay is used to examine the extract’s ability to promote the development and migration of healthy cells into a wound, thereby encouraging its healing. Adhesion and invasion of *S. lugdunensis* (a harmless skin commensal that could become a life-threatening pathogen) evaluate the extent to which binding and penetration into deeper layers of cells would be avoided in areas treated with the plant extract. However, before considering any direct application of the plant extract, cytotoxicity testing should be conducted first, as it demonstrates whether it is safe to apply to human skin.

Given the foregoing, as well as the fact that flowers of herbaceous peonies have received insufficient attention for their valuable constituents, the goal of this study was to reveal the chemical profile of different extracts from the petals of *Paeonia tenuifolia* L. originating from various geographical regions in the Republic of Serbia, and to examine their effects in a number of assays related to human skin healing. 

## 2. Results

### 2.1. Chemical Profile 

To the best of our knowledge, this is the first study exploring the chemical profile of the petals of *P. tenuifolia* growing wild in Serbia. In the examined methanolic extract of the petals collected in Gulenovci, **83** compounds were identified and further classified into the following major groups (Table S1): phenolic acids (compounds **1**–**39**), flavonoid glycosides and aglycones (compounds **40**–**58**), anthocyanins and anthocyanidins (compounds **59**–**69**), terpene derivatives (compounds **70**–**80**), and other compounds (**81**–**83**).

*Phenolic acids*. This is the most abundant group, with 39 identified compounds. For the positive identification of compounds **3**, **29**, and **36**, chromatographic characteristics matching the standards of gallic acid, ellagic acid, and p-coumaric acid were used, respectively. Phenolic acids **3**, **29**, **36**, and **38** were previously identified in *Paeonia* taxa, while compounds **10**, **16**, **24**, **32,** and **37** are tentatively detected for the first time in the petals of *P. tenuifolia* in this study. Gallic acid (**3**) and ferulic acid (**38**) were formerly identified in the roots of the herbaceous *Paeonia lactiflora* [24,25]. Ellagic (**29**) and p-coumaric acids (**36**) were found in the stamens of the tree *Paeonia ostii* [26]. It should be noted that the quantification of these compounds has been undertaken (Table S2), and the amount of *p*-coumaric acid was in the range of 0.253–15.04 mg/L, except for the maceration methanolic extracts of the petals from Gulenovci and the maceration and UAE methanolic extracts of petals from Deliblato sands, where it was not identified. On the other hand, ellagic acid was found in all of the analyzed extracts, and its content varied from 30.67 to 166.33 mg/L (the highest concentration was discovered in the aqueous maceration extract of the petals collected in Gulenovci). Compound **37** (hydroxybenzoyl-galloyl-hexoside) was observed at 6.44 min with a molecular ion at 451 *m*/*z*. It gave its MS^2^ base peak at 313 *m*/*z* formed by the loss of 138 Da (hexosyl). Its MS^3^ base peak was at 169 *m*/*z* (loss of galloyl group—144 Da), while its MS^4^ base peak was generated by further loss of CO_2_ (18 Da). Compounds **19** (3.70 min), **23** (4.07 min), **30** (5.19 min), and **33** (5.74 min) were identified as derivatives of digallic acid, and their fragmentations were recently confirmed [27,28]. Compounds **13** (3.29 min), **15** (3.49 min), and **21** (4.02 min), with the same accurate masses (483 *m*/*z*) and similar fragmentation patterns, were found to be isomers of digalloyl-hexoside. The MS^2^ base peak in all three cases was found at 169 *m*/*z*, which may correspond to either digallic acid residue or hexose residue. Five other compounds, **1** (0.57 min), **2** (1.22 min), **4** (1.78 min), **5** (2.13 min), and **9** (3.17 min), with the same exact masses (331 *m*/*z*) and similar fragmentation patterns, are assessed to be the isomers of galloyl-hexoside. For all of them, the MS^2^ base peak was observed at 125 *m*/*z*, which may correspond to residues of gallic acid or hexose. Similar fragmentation patterns have also been found to be isomers of tetragalloyl-hexoside, within compounds **26** (4.82 min), **28** (4.95 min), and **31** (5.22 min), which are characterized by the same accurate masses (787 *m*/*z*). For all of them, the MS^2^ base peak at 617 *m*/*z* was detected, which is probably due to the tetragallic acid or hexoside residue. Regarding the remaining compounds presented in Table S1, isomers of dihydroxybenzoyl-hexoside (**6**, **11**), dihydroxybenzoic acid (**7**, **25**), and galloyl-di-O-hexoside (**8**, **12**) were also identified, and their MS^2^ peaks were 153, 109, and 313 *m*/*z*, respectively. The other ten compounds from the phenolic acids group (**14**, **17**, **18**, **20**, **22**, **27**, **34**, **35**, and **39**) were identified based on their characteristic MS spectra and fragmentation patterns. 

*Flavonoid glycosides and aglycones*. This is the next most numerous group of compounds, with 19 compounds identified in the examined extract. Compounds **41** and **43** were recently identified in the seeds of the herbaceous *P. lactiflora* [29]. In the roots of herbaceous peonies, compounds **54** in *P. lactiflora* and *Paeonia veitchii* [30] and **56** (onopordin) in *P. anomala* [31] were detected. Apart from the roots, compounds **47** and **57** were also identified in the aerial parts of *Paeonia kesrounansis* [32] and *Paeonia parnassica* [33]. Previously, only tree peonies (several cultivars) were found to contain compounds **42**, **45**, **48**, **49**, and **52** [34], while compounds **44**, **46**, **51**, **53**, **55**, and **58** were detected earlier in the flowers of herbaceous *Paeonia delavayi* or tree *P. rockii* [35]. The compounds **55** and **58** were quantified, and the amount of compound **55** varied from 0.159 to 3.15 mg/L (the highest amount was found in the maceration methanolic extract of petals from Deliblato sands), while isorhamnetin was present in amounts ranging from 0.26 to 7.22 mg/L (the highest content was detected in the UAE aqueous extract of the petals from Pančevo). 

*Anthocyanins and anthocyanidins*. In the presented study, 11 compounds were identified. The following seven were already discovered in the *Paeonia* taxa. In tree peonies, only two (**61**, **62**) were found in *P. delavayi* [36]. However, most of them were reported in the flowers of herbaceous peonies, namely **59** and **63** in *P. peregrina* and *P. lactoiflora* [37,38], **60** only in *P. lactiflora* [39], and **67** and **68** only in *P. tenuifolia* [40]. The anthocyanidins, delphinidin (**67**) and cyanidin (**68**), were also identified in the petals of some tree peonies, namely *Paeonia jishanensis*, *P. ostii*, *P. ostii* var. *lishizhenii*, *Paeonia qiui*, *P. rockii*, *Paeonia decomposita*, *Paeonia potaninii,* and *P. delavayi* [35]. 

*Terpene derivatives*. Similar to the previous, 11 compounds from this group were detected, and all of them were already reported as characteristic for the *Paeonia* taxa. In herbaceous peonies, compounds **77**–**80** were detected in all plant organs [41], compounds **70**–**73** only in the roots of *P. lactiflora* [24,41], **70**–**71** also in the roots of *P. veitchii* [24], **78** in the roots of *Paeonia daurica* [42], and **74**–**76** only in the flowers of *P. tenuifolia* [43]. Several compounds identified in this study were also previously reported in tree peonies; **74**–**76** in the stamen of *P. ostii* [26] and **77**–**80** in all plant organs of *Paeonia suffruticosa* [44]. In this study, molecular ions of isomeric compounds **77**, **79**, and **80** (7.55, 8.27, and 9.36 min, respectively) were found at 629 *m*/*z*, and their fragmentation patterns were very similar. Tentative identification of these three compounds, precisely benzoyl paeoniflorin + HCOOH 1 (**77**), benzoyl paeoniflorin + HCOOH 2 (**79**), and benzoyl paeoniflorin + HCOOH 3 (**80**), was achieved by their chromatographic characteristics [44].

*Other compounds*. In this study, following three compounds were also tentatively identified for the first time in the petals of *P. tenuifolia*: shikimic acid (**81**), citric acid (**82**), and pinoresinol hexoside (**83**). All of them were previously reported in the *Paeonia* taxa, but none of them as constituents of the petals. Compound **82** was previously detected in the roots of herbaceous *P. lactiflora* and *P. veitchii* [24], while in tree peonies, compound **81** was found in the plantlets of *P. suffruticosa* [45] and compound **83** in the stamens of *P. ostii* [26]. 

### 2.2. UV-Vis Chemical Analysis 

*Total polyphenol content (TPC)*. TPC values determined for the extracts of *P. tenuifolia* petals collected at various localities in Serbia are presented in Table 1. 

Both the extraction process and the extraction medium had a significant impact on the TPC values, which also varied depending on the origin of the plant material. When water was used as an extraction medium, the highest TPC values were detected in the petals from Deliblato sands (33.26 and 32.94 mg GAE/g for the extracts from MAE and UAE, respectively). The TPC of the mentioned extracts was significantly higher in comparison to the maceration extract and extracts of the petals from Gulenovci and Pančevo. In the water extracts of petals from Gulenovci and Pančevo, the highest TPCs were observed when maceration was employed (31.34 and 24.04 mg GAE/g, respectively), while the TPC value for Gulenovci samples was significantly higher compared to Pančevo parallels. Additionally, in the Gulenovci and Pančevo samples, the UAE and MAE have given significantly lower polyphenol yields. When methyl alcohol (MeOH) was used as an extraction medium, the highest TPC values were observed using the MAE method for the petals from Gulenovci and Pančevo (35.24 and 28.15 mg GAE/g, respectively) and the UAE method for the Deliblato sands’ petals (32.83 mg GAE/g). When methanol was used as an extraction solvent, Gulenovci petals extracts had a higher TPC in comparison to those from the other two origins.

*Total flavonoids content (TFC)*. TFC values determined for the extracts of *P. tenuifolia* petals collected at various localities in Serbia are also presented in Table 1. When water was used as an extraction medium, the MAE proved to be the most suitable extraction technique for all three localities in Serbia; the petals from Deliblato sands showed the highest TFC (28.48 mg CE/g), which differed not only from the petals from Gulenovci (23.09 mg CE/g) and Pančevo (16.78 mg CE/g), but from all other extraction methods. However, the trend for methanolic extracts was somewhat different. When methyl alcohol was used as an extraction medium, the highest TFC was detected in the macerated petals of Gulenovci (24.50 mg CE/g), which did not differ from the highest TFC obtained by MAE of the petals from Pančevo (19.73 mg CE/g) but differed from the highest TFCs obtained by UAE and maceration of the petals from Deliblato sands, which did not differ between themselves (23.72 and 16.01 mg CE/g, respectively).

### 2.3. Antioxidant Activity 

The results of four antioxidant assays conducted with *P. tenuifolia* extracts of petals collected at various localities in Serbia are presented in Table 2. 

***Free radical scavenging activity.*** As both the DPPH and ABTS assays present the ability of the tested extracts to scavenge free radicals and reduce them into an inactive form, the obtained results could be observed simultaneously. In this study, when water was used as an extraction agent, the extracts with the highest DPPH^●^ radical scavenging activity (the lowest IC_50_) did not differ significantly between the regions from which the petals were collected. In addition, the strongest ABTS^●^ reducing activity of the aqueous extracts was obtained for the extracts acquired by the UAE in all three geographical localities, while for the Deliblato sands, the MAE extract showed the same activity as the UAE. In the case of methanolic extracts, the DPPH scavenging activity was the highest for the Deliblato sands and Gulenovci MAE extracts (0.125 and 0.123 mg/mL, respectively), whereas for Pančevo, the best activity was for the extracts obtained by maceration (0.125 mg/mL), but none of the methanolic extracts, regardless of the extraction process, differed amongst themselves. For the ABTS scavenging activity, the most efficient were the MAE extracts from Gulenovci and Deliblato sands (0.098 and 0.097 mg/mL, respectively), while for the extract from Pančevo, maceration proved to be the most efficient method, though the values did not differ significantly. In addition, when methyl alcohol was used as an extraction solvent, the extracts of petals from Gulenovci and Deliblato sands that were most efficient in the reduction of both types of free radicals were acquired by the same extraction method (maceration for the ABTS assay and MAE for the DPPH).

***Ion reducing antioxidant activity.*** The reducing power assay is commonly used to evaluate an antioxidant’s ability to donate an electron, which is a key mechanism of phenolic antioxidant action. The obtained CUPRAC reducing values significantly differed when petals from different geographical areas and different extraction techniques were used. The water samples that showed the most satisfactory CUPRAC reducing values were the MAE extracts of petals from Pančevo and Deliblato sands (0.385 and 0.391 mol TE/g, respectively) and the macerated one of the petals from Gulenovci (0.386 mol TE/g). When methanol was used as a solvent, the MAE method was found to be the most efficient for the petals of all three origins, which differed significantly amongst themselves (the CUPRAC values for the MAE extracts of petals from Gulenovci, Pančevo, and Deliblato sands, respectively, were 0.358, 0.367, and 0.354 mol TE/g). The results of the FRAP assay for the aqueous extracts showed that the UAE method gave the best results for the petals from all three localities (843.39, 832.4, and 815.58 μmol Fe^2+^/g, for Gulenovci, Pančevo, and Deliblato sands, respectively). When methanol was used as a solvent, the most efficient were the MAE extracts from the petals of Gulenovci and Deliblato sands (840.46 and 833.88 μmol Fe^2+^/g, respectively), and the UAE extract from Pančevo (777.16 μmol Fe^2+^/g), and they differed significantly. 

In the majority of cases when water was used as an extraction agent, UAE proved to be the best method, as it gave more favorable results for most of the antioxidant activities, while in the majority of cases when methyl alcohol was used as an extraction agent, MAE showed to be the most suitable extraction method.

### 2.4. Antimicrobial and Antibiofilm Activities

Results of the antibacterial efficacy of the aqueous and methanolic extracts of *P. tenuifolia* petals, collected from different regions of Serbia and tested against the following three bacteria: *Staphylococcus lugdunensis*, *Staphylococcus aureus*, and *Proteus vulgaris*, are presented in Table 3. 

The antibacterial activity of the extracts of *P. tenuifolia* petals from Gulenovci showed the best antibacterial activity, with the extracts being the most effective against S. lugdunensis (MIC 0.125–1 mg/mL), followed by P. vulgaris. The bacteria that showed the most resistance to the influence of the extracts of petals from this locality was S. aureus (MIC 0.25–2 mg/mL). On the other hand, the extracts of the petals from Pančevo and Deliblato sands showed similar activity against all three bacteria, with MIC values of both groups of extracts against S. lugdunensis varying from 0.5–1 mg/mL, and thus being the most active in inhibiting its growth. Additionally, methanolic extracts of the petals from all three localities in Serbia showed better activity against S. aureus (MIC 0.25–1 mg/mL) in comparison to the positive control, the antibiotic gentamicin (MIC 1.33 mg/mL).

The extract’s antifungal activity was tested against three medically important Candida species (Table 4). The efficacy of each extract was compared with that of ketonazole, the standard antifungal agent. All of the *P. tenuifolia* petal extracts used in the study had varying degrees of antifungal activity against the Candida strains. However, the most notable antifungal effect was achieved against Candida kefyr, an important fungal pathogen capable of causing invasive candidiasis in immunocompromised patients, particularly those with oncohematological diseases. The extracts’ MIC values against C. kefyr ranged from 0.5–1 mg/mL, whereas the MFC values ranged from 1–2 mg/mL. C. krusei was the most resistant, with a MIC value of 2 mg/mL and a MFC value of 4 mg/mL. The antifungal activity of the tested extracts decreased in the following order: Candida kefyr > Candida albicans > Candida krusei.

**Figure 1:**
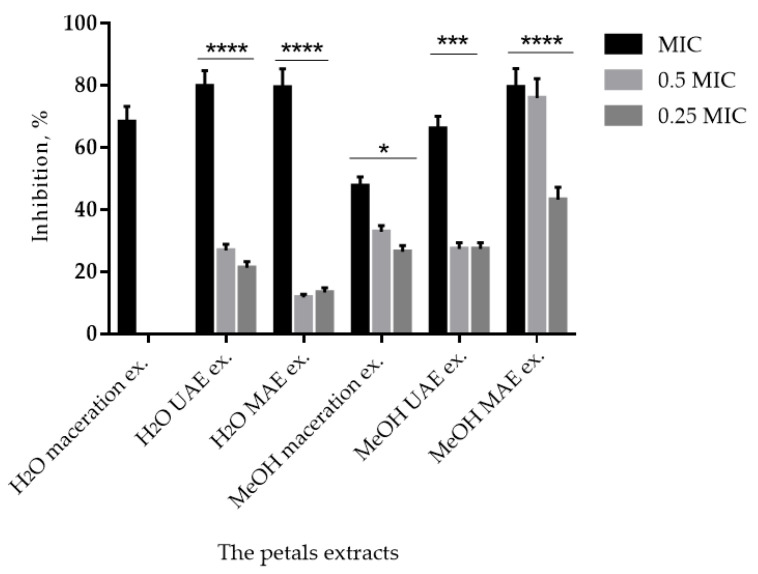
Biofilm formation of S. lugdunensis (Ibis 2996) after treatment with *P. tenuifolia* extracts. The asterisks represent statistical significance: * *p* ≤ 0.05; ***, *p* ≤ 0.001, ****, *p* ≤ 0.0001.

Since the extracts of the petals from Gulenovci showed the best inhibitory activity against *S. lugdunensis*, they were chosen for further testing of their antibiofilm activity and the results are presented in Figure 1. In particular, the methanolic extract of the petals from Gulenovci that was acquired by the MAE method showed an inhibition of biofilm formation by 79% at an MIC value, decreased to 76% at ½ MIC value, and was 43% at ¼ MIC value. For the aqueous extracts, the one obtained by the UAE method showed the highest ability to inhibit the biofilm formation by S. lugdunensis, with 80% inhibition at the MIC value.

### 2.5. Cytotoxicity of Paeonia tenuifolia Petals Extracts 

The growth rate of the cell line was not adversely affected by any of the petal extracts. The findings showed that none of the extracts had any cytotoxic effect, as their IC_50_ values were greater than 400 µg/mL.

### 2.6. Wound Healing

**Figure 2:**
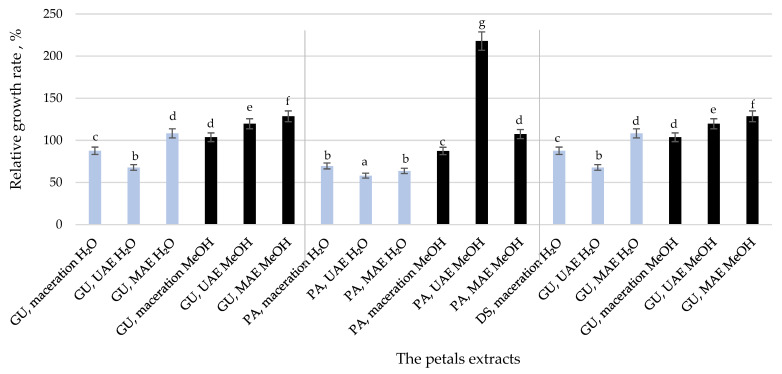
Relative growth rate of the HaCaT cell line in the presence of *P. tenuifolia* extracts. The letters represent statistical significance: a *p* ≤ 0.05; b and c, *p* ≤ 0.001; d and e, *p* ≤ 0.0001; e and f, *p* ≤ 0.00001; g, *p* ≤ 0.000001.

Wound healing is an essential part of the skin’s defensive and protective functions [46]. Based on the results for the keratinocytes’ proliferation under the influence of the petal extracts (Figure 2), only those that showed a higher rate of cell growth compared to the control were included in the wound healing assay. Table 5 presents the effects of the extracts on the wound closure compared to the control (an untreated wound). 

The application of the aqueous MAE extract of *P. tenuifolia* petals from Gulenovci to HaCaT cells with scratched wound gaps resulted in a notable improvement in the wound gap closure compared to the control. In the case of methanolic extracts, the one obtained by maceration of the petals from Deliblato sands proved to be the best, yet it was less effective compared to the aqueous extract (by 26.6%). With regard to the achievement of the remaining extracts (Table 5), the UAE methanolic petal extract from Gulenovci showed some efficacy compared to the control, and it was stronger than the MAE methanolic extracts from Pančevo and maceration from Gulenovci, whose influences on cell migration were similar.

### 2.7. Adhesion and Invasion Capacities to HaCaT Cells by Staphylococcus lugdunensis

**Figure 3:**
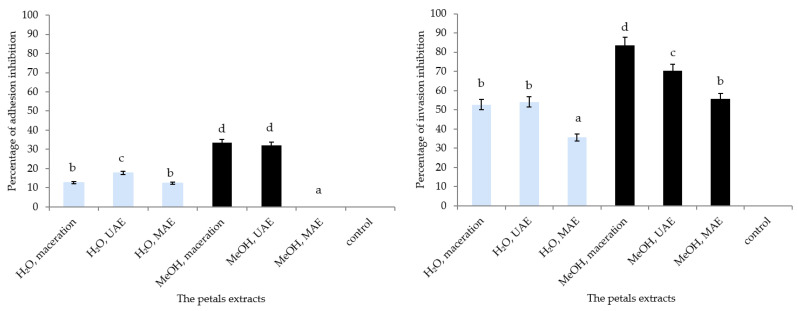
*S. lugdunensis* adhesion (**left**) and invasion (**right**) capacities to HaCaT cells treated with selected *P. tenuifolia* extracts; presented as percentage of adhesion and invasion compared to untreated cells (control). The letters represent statistical significance: a, *p* ≤ 0.05; b, *p* ≤ 0.001; c, *p* ≤ 0.0001; d, *p* ≤ 0.00001.

Extracts of the petals collected in Gulenovci were chosen for further study as they proved to be the most effective in inhibiting the growth of *S. lugdunensis* (Table 3). The effects of six extracts from the petals originating from this locality on their ability to inhibit *S. lugdunensis* adhesion and invasion of HaCaT cells showed that both invasion and adhesion were markedly reduced by all extracts, except the methanolic MAE one, which did not show any adhesion inhibition (Figure 3). The aqueous extracts showed a lesser inhibition of bacterial adhesion than 20%, whereas the maceration and UAE methanolic extracts were more efficient, with 33% and 32%, respectively. The effect of the extracts on *S. lugdunensis’* invasion ability was more profound; more than 50% inhibition was obtained by the methanolic MAE extract, gradually increasing, and being highest for the methanolic maceration petal extract (83.7%).

## 3. Discussion

### 3.1. Chemical Composition of the Petal Extracts Determined by UV-Vis Spectrometry 

When all the values for TPC are taken into account, methyl alcohol appears to be a better extraction medium for the extraction processes employed in this study. However, there were two exceptions in which the water as a solvent dominated over the methyl alcohol: the MAE extract of petals from Deliblato sands and the macerated extract of petals from Pančevo. In short, methyl alcohol is considerably less polar than water, thus allowing an easier extraction by dissolving more of the phenolic compounds from plant material [47]. In addition, for methyl alcohol, it has already been proven that it easily extracts phenolic acids and catechin [48]. The deviation that occurred during the maceration of the petals collected in Pančevo can be attributed to the content of some non-phenolic compounds (carbohydrates or terpenes) in the extract or to the mutual formation of a complex of certain compounds [49]. The highest TPC values were anticipated when the UAE method was employed because the cavitation process that occurs during sonication causes the plant cell walls to swell and rupture, allowing either higher rates of diffusion through the cells or washing out of the content [50]. The fact that this was observed only in the case of the petals from Deliblato sands could be explained by their different chemical composition; it is possible that there are certain chemical compounds in the petals that are extractable only with methyl alcohol when the UAE method is used. 

When methyl alcohol was used as a solvent, the best results were achieved by the MAE method, which could be attributed to the ability of microwaves to penetrate the cell walls of the plant material and interact with the polar molecules, further leading to volumetric heating of the biomaterial and an increase in pressure inside the cells, causing the rupture of the cell walls and the release of phenolic analytes [51]. This explains the obtained TPC values in this study. Additionally, the higher TPC values could be produced by the decomposition of larger phenolic compounds into smaller ones while retaining their original characteristics, as assessed by the Folin–Ciocalteu assay [52]. Higher TPC values of aqueous extracts obtained by maceration than those acquired by the MAE process from the petals of Gulenovci and Pančevo could be attributed to the degradation of phenolic compounds that the Folin–Ciocalteu assay was unable to detect [53]. Apart from this, the TPCs may have decreased not only as a result of interactions between phenolic and non-phenolic compounds (sugars, fatty acids, etc.), but also as a result of the oxidation process during the extraction procedure [54]. There are very few studies in the literature that report the TPC in the petals of herbaceous peonies. In several cultivars of *P. lactiflora* Pall., TPC of 11.16–32.23 mg/g was detected [55,56,57], while in *P. peregrina* Mill. var. *romanica,* a high TPC content was detected (642.03 mg GAE/g) [58]. The TPC content in ethanolic extracts of the petals of *P. lactiflora* obtained by the MAE method was 54.45 mg GAE/g and by the UAE method, 83.16 mg GAE/g, both values being notably higher compared to the TPC in our extracts. This could be attributed to the use of a different extraction solvent and higher extraction temperatures [59]. As tree peonies have been investigated more extensively, it could be interesting to note that the TPC values of the methanolic extracts of the petals of various cultivars of *P. delavayi* ranged from 388.35 to 469.4 mg GAE/g [60], while in aqueous petal extracts of cultivars of *P. suffruticosa,* it ranged from 23.94 to 130.78 mg GAE/g [61]. The results from this study were the most similar to that of a *P. suffruticosa* hybrid with white petals, known as ‘Danfeng Bai’ [61].

The lower TFC contents in MAE methanolic extracts than in MAE water extracts were attributed to the flavonoids’ richness in hydroxyl groups, which provide them with higher solubility in less polar solvents. In most cases, the increase of water content in the extraction solvent increased the MAE efficiency, possibly because the additional water caused the plant material to swell more, which enlarged the contact area between the solvent and the plant matrix [62,63]. Because flavonoids are not very stable and interact with other compounds in the food matrix, it was assumed that the TFC bioaccessibility correlates with its chemical characteristics [64]. Few studies have been conducted on the TFC of the petals of herbaceous peonies. The TFC value of 123.48 mg QE/g for the ethanolic MAE extract [59] was much higher than those obtained for both the methanolic and aqueous extracts of the petals of *P. tenuifolia* in this study, which could be attributed to possible differences in the chemical composition of the petals of different *Paeonia* species. 

### 3.2. Antioxidant Activity of P. tenuifolia Extracts 

An antioxidant is a chemical compound or plant extract that can delay or limit the oxidation of lipids or other molecules by reducing the onset or propagation of oxidative chain reactions, thereby preventing or repairing damage caused by oxygen to the body’s cells. It functions through one or more of the following mechanisms: reducing activity, free radical scavenging, possible complexing of pro-oxidant metals, and singlet oxygen quenching [65]. The assays performed in this study function through free radical scavenging and reducing activities. In detail, the mechanism of action of the extracts with observed ABTS or DPPH free radical-scavenging activity was as a hydrogen donor, and it terminates the oxidation process by converting free radicals to more stable products. Further, the extract with a positive FRAP or CUPRAC assay result functions as an electron donor, and it terminates the oxidation chain reaction by reducing the oxidized intermediates into the stable form [65]. However, depending on the test used, the chemical complexity of the extracts could end up with inconsistent results, and that is why it is strongly advised to conduct the screening using numerous assays [66,67,68].

The aqueous extracts of the petals from Gulenovci and Pančevo showed the highest DPPH^●^ radical scavenging activity when the UAE method was employed, followed by MAE, making the maceration process the least favorable method for this assay. Even though the TPC of the aqueous extracts was the highest for those obtained by maceration, the DPPH assay proved to have the lowest radical scavenging activity. In a previous study using extracts from another plant, there was a negative correlation between the total number of phenolic compounds and the outcomes for scavenging DPPH radicals [69]. The authors have indicated that some phenolic compounds that function as antioxidants and react with Folin–Ciocalteu’s reagent might not react with the DPPH free radicals. Additionally, phenolic compounds in their free form may be scavenged by the DPPH free radical, whereas Folin–Ciocalteu’s reagent measures phenolics in either their free or bound forms [70]. The incidence of the lowest IC_50_ value for DPPH scavenging activity in the aqueous extracts of the petals of *P. tenuifolia*, which had the lowest TPC values observed in this study, could be explained by the ability of other compounds from the extracts to reduce the DPPH^●^ radical. This is in accordance with findings from a previous report in which 223 herbal infusions showed a very poor correlation between antioxidant activity and the TPC values [71]. Additionally, a negative association between the TPCs and antioxidant activity was also observed in the herbal tea of *Cosmos caudatus* [72]. Thus, apart from the phenolic molecules, other phytochemical compounds also contribute to the antioxidant activity of plant material [73]. The DPPH^●^ radical scavenging activity in the ethanolic petal extracts of *P. lactiflora* hybrid ranged from 70 to 79% [59], in the methanolic extracts of purple and yellow petals of *P. delavayi* it was 324.24 and 336.08 μg of Trolox/mg, respectively [60], whereas in the aqueous extracts of different colored petals of *P. suffruticosa* cultivars, it ranged from 35.47 to 130.78 mg TE per g [61]. Although the difference in the ability to scavenge DPPH^●^ radicals could possibly be attributed to the different chemical composition of the petal extracts from the different peony species that were mentioned, none of the presented results could be compared among themselves or with the corresponding ones in this study due to the differences in the way those results were expressed.

The antioxidant capability of various compounds (antioxidants) on nonphysiological free radicals (produced by smog, pesticide pollution, cigarette smoke, and chemical reagents) could be indirectly characterized by ABTS free radical scavenging ability [74]. The antioxidant activity of the petal extracts examined in this study showed significant differences, which was to be expected since the IC_50_ values ranged from 0.070 to 0.099 mg/mL. The highest ABTS antioxidant capacity obtained when the UAE method was employed and water was used as a solvent might be explained by the fact that the increase in temperature probably caused the enhanced solubility of analytes in the extraction solvent and their better diffusion rate from the solid matrix [75,76]. In addition, the ultrasound waves influence the extraction of bioactive compounds by decreasing the pressure of the liquid, which happens as a result of the expansions. When the pressure exceeds the tensile strength of the liquid, vapor bubbles form and implosively burst due to cavitation, which is caused by strong ultrasonic fields [77]. Microporous biomass particles are disturbed by macroturbulence, high velocity interparticle collisions, and macroturbulence as cavitation bubbles pop. In near liquid–solid interactions, cavitation causes a fast-moving stream of liquid to be directed through the cavity at the surface [78]. The impingement of these microjets results in surface peeling, erosion, and particle breakdown, which facilitates the release of bioactives from the biological matrix. This impact enhances extraction efficiency by increasing mass transfer via eddy and internal diffusion mechanisms [79,80]. All of this could have contributed to an increase in the ABTS^●+^ radical scavenging activity. On the other hand, the results from this study showed that for the localities of Deliblato sands and Gulenovci, the lowest IC_50_ values were for the extracts obtained by MAE with methyl alcohol as a solvent, which is in agreement with the results of Mohapatra et al. [81] who also suggested phenolic compounds as the primary antioxidants in the plant extracts. These may work as antioxidants in a variety of ways, such as radical scavengers, reducing agents, hydrogen donors, and metal chelators [82]. The presence of some non-phenolic compounds, such as ascorbates, carotenoids, and terpenes, which may also contribute to the overall antioxidant activity, can explain why the incidence of the ABTS^●+^ radical scavenging ability was higher when the TPC was lower [83]. As the petals of the endangered herbaceous peonies have been under-investigated, there are very few studies that have assessed their antioxidant capacity on the basis of ABTS^●+^ free radical scavenging activity. The ABTS^●+^ radical scavenging activity of the petals of *P. lactiflora* was 30.14 μmol Trolox/g [61], whereas in the ethanolic extract of the petals of *P. lactiflora* hybrid, it ranged from 75 to 83% [59]. Although the petals in our study originated from the petals of different peony species, they could not be compared with the ones aforementioned due to the differences in the way the results were expressed.

The results of the CUPRAC assay varied in a wide range, and such a phenomenon could potentially be explained by the release of different chemical compounds that are present in the petals and which are responsible for the extracts’ ability to reduce the cupric ion. Epicatechin gallate, epigallocatechin gallate, quercetin, fisetin, catechin, caffeic acid, epicatechin, gallic acid, rutin, and chlorogenic acid were compounds previously reported to exhibit the greatest cupric ion-reducing capacity, due to the position and number of the hydroxyl groups in the molecule, and the degree of conjugation inside the entire molecule that facilitates simple electron transport [84]. The deviation that has occurred in the aqueous petal extract from Gulenovci, where the highest antioxidant capacity was not associated with the highest TPC values, might be explained by the fact that certain phenolic compounds do not react with the Al^3+^ present in the AlCl_3_ salt used in the TFC test and thus cannot be quantified by this colorimetric analysis [85]. Fundamentally, flavones and flavonols react with Al(III) while flavanones and flavanonols do not complex to the same degree. Some flavones and flavonols that react with Al(III) are quercetin, myricetin, morin, and rutin [85]. The CUPRAC merits are generally higher for the aqueous than for the methanolic extracts, which can be attributed to the difference in solubility of certain chemical compounds in these solvents [86]. A variety of antioxidants, including polyphenols, flavonoids, thiols, D-ascorbic acid, mannitol, glucose, and others, can lower cupric ions [87]. Additionally, specific environmental factors, such as climate, locality, and temperature, might significantly impact the accumulation of compounds that carry antioxidant potential [88], which could also be used to explain differences in antioxidant capacity between the samples of the same plant species collected at different geographic locations. A thorough review of the literature revealed that the CUPRAC method had never been employed to measure the antioxidant activity of petals from any peony species. 

An important characteristic of the FRAP assay method is that it estimates the antioxidant capacity of only hydrophilic components of the extracts and is not accurate for components that are more soluble in organic solvents, such as alcohols [89]. This could explain the highest FRAP value observed in the aqueous UAE from the petals collected in Gulenovci. The deviations in FRAP values of the methanolic extracts from Gulenovci and Pančevo, where the MAE extract was more efficient in antioxidant activity than the UAE extract, can be explained by the fact that the temperature increase likely promoted the efficiency of the MAE as a result of the increased diffusion rate and better solubility of compounds in the solvents; a similar finding was previously reported by Dorta et al. [90]. To the best of the authors’ knowledge, there are no previous studies on the FRAP antioxidant activity of the petal extracts of *P. tenuifolia*. The FRAP value for the petal methanolic extract of *P. lactiflora* was 257.2 mg ascorbic acid/100 g [58], which cannot be compared to the results from this study, as the calibration curves were made on the basis of different compounds.

### 3.3. Antimicrobial and Antibiofilm Activities of P. tenuifolia Extracts

According to Rios and Recio [91], phenolics are the primary group of compounds of plant origin associated with antimicrobial activity. Their microbicidal activity is particularly attributed to the position and number of the hydroxyl groups in the benzoic ring of phenolic acids. As the number of OH groups increases, so does the activity, which can be translated into lower inhibitory and bactericidal concentrations [92].

Herein, aqueous and methanolic extracts of the petals of *P. tenuifolia* were studied as a potential source of antimicrobial agents intended for use in the therapeutic treatment of human skin. As the phenolic acids represented the most abundant group of compounds in the analyzed extracts, with the dominance of gallic acid and its derivatives, which have already been confirmed as strong antibacterial agents [93,94], such activity was expected. The extracts of the petals from Gulenovci had the highest antibacterial activity against *S. lugdunensis*, followed by *S. aureus* and *P. vulgaris* (Table 4), which required a higher extract concentration for the inhibition of bacterial growth. The extracts of the petals from Pančevo and Deliblato sands showed a lower ability to inhibit the degree of bacterial growth, both being the most effective against *S. lugdunensis*. The antibacterial activity of the *Paeonia* taxa has been investigated in the past. The methanolic UAE extracts of the fruits of the tree peony *P. rockii*, which are also rich in gallic acid, showed satisfactory activity against *S. aureus* and *P. vulgaris* (the assay was performed by the disc diffusion method, and the results varied between 6 and 14 mm) [26], which is in agreement with our results. Contrary to our findings, in the same study [26], the methanolic extracts had a greater capacity to prevent the bacterial growth of *P. vulgaris* and *S. aureus*, which could be attributed to the difference in chemical composition between the studied peony species. The MIC and MBC values for the petal extracts in this study were much higher than those in the positive control (antibiotic), which is not surprising, as it is well known that the extracts are complex mixtures of many compounds, and the concentration of each active compound is likely much lower. However, as gentamicin intended for topical uses may adversely affect osteogenesis [95], cause nephrotoxicity [96], etc., “natural pharmacons” appear to have a bright future as they have fewer side effects and are more available. 

Fungal pathogens are not an exception in the age of antimicrobial resistance, which is why a number of recent studies have been investigating the efficacy of antifungal plant compounds [97]. In this paper, we look at the use of natural compounds present in the aqueous and methanolic petal extracts of *P. tenuifolia* to fight fungal infections caused by the most common human fungal pathogens, *Candida albicans*, *C. kefyr,* and *C. krusei*. The medicinal value of plants is derived from the various chemical constituents, and the components that are proven to exhibit antifungal activity are various phenolic compounds, such as gallic acid, thymol, flavonoids, particularly catechin, polyphenols such as tannins, terpenoids, and saponins. As all of the petal extracts contain gallic acid, the antifungal assays reasonably showed satisfactory activity; the extracts were most effective in inhibiting *C. kefyr*, followed by *C. albicans*, and were the least efficient in controlling *C. krusei*. Similar to the antibacterial activity, the petals collected in Gulenovci showed the highest antifungal capacity, as they had the lowest MICs and MFCs for all the tested microfungi. This could be attributed to the difference in chemical composition between the extracts, as all of the aqueous extracts of the petals from Gulenovci had the highest content of ellagic acid amongst all of the extracts, while for the methanolic ones, the amount of this compound was notably lower. Ellagic acid belongs to phenolic acids, which have been found to be weak acids with the ability to dissociate a cell membrane, thus exhibiting antimicrobial activity [98]. Previous work has been conducted on the effects of some extracts from *Paeonia* taxa on various fungal pathogens. Picerno et al. [99] presented the root extracts of the tree peony *P. rockii* as very efficient (MIC_50_ = 30 μg/mL) against *C. albicans*, and it was by 88% more efficient than the extracts of the petals from Gulenovci in this study. On the other hand, Yuan et al. [100] examined the effects of the extracts of the roots of seven populations of the herbaceous *P. veitchii* on *C. albicans*, and the results were in agreement with ours (MICs 0.25–1 mg/mL). The most dominant constituents of the root extracts were also observed in all of the petal extracts examined in this study. 

Plant extracts high in polyphenols have previously demonstrated antimicrobial activity, as well as a reduction in biofilm formation and adhesion of *S. lugdunensis* and other bacterial species to artificial surfaces and epithelial cells [101]. The outcomes of this study (Table 4 and Table 5) showed that the extracts of the petals from Gulenovci had the highest activity against all of the tested pathogens and the most efficacy in inhibiting the growth of *S. lugdusnensis*, which were the major reasons why they have been further studied on the biofilm formation (Figure 1). Among the methanolic extracts, the one obtained by MAE showed the best potential, and was 17% greater than that of the UAE and 37.5% greater than that of the maceration. Among the aqueous extracts, the one acquired by UAE revealed the highest antibiofilm formation activity, was almost identical to the activity of MAE, and was 15% greater than the extract made by the maceration method. Keeping in mind that the extracts with the highest activity, namely the methanolic MAE and the aqueous UAE, showed almost identical suppression of *S. lugdunensis* biofilm formation, both solvents could be equally successfully used to extract bioactive constituents from the petals of *P. tenuifolia* responsible for this type of activity. As the amounts of the quantified compounds varied significantly between the two extracts that showed the highest biofilm formation inhibition, it is possible that certain compounds that were not quantified contributed to this type of activity. To the best of the authors’ knowledge, no previous work has undertaken done on the effects of herbaceous peonies on bacterial biofilm formation. 

### 3.4. Cytotoxicity and Wound Healing Capacity of the P. tenuifolia Petals Extracts

The impact of any plant extract on cell metabolism and viability is crucial for determining the extract’s safety and toxicity. As the focus of this study was the assessment of the cytotoxic properties of the petal extracts on keratinocytes (the predominant cell type in the outermost layer of the skin, called the epidermis), the obtained results might be considered promising as they presented primarily the absence of cytotoxicity; no toxic effect on HaCaT cells was observed. In addition, while all of the extracts showed a similar effect on the viability of HaCaT cells, some of them also exhibited proliferative properties, while some others decreased cell viability (Figure 2). The positive or negative effect of the petal extracts on cell proliferation may be associated with high concentrations of single phenolic and flavonoid compounds [102]. All of the extracts were rich in phenolic acids and flavonoid glycosides and aglycones, but those that were shown to be the most efficient in aiding the cells growth had, in most cases, a lower amount of *p*-coumaric acid than those that inhibited growth. After a detailed literature review, the authors found that no previous work has been undertaken on the cytotoxicity of the petal extracts of herbaceous peonies on human keratinocytes. 

Because of their low development costs and minimal side effects, the skin healing benefits of plants and their extracts have been intensively studied with the aim of producing agents that stimulate wound healing [103,104]. In this study, only the extracts that showed a positive effect on cell growth (Figure 2) were further subjected to the wound healing assay. The possible mechanisms by which the extracts aid wound healing include maintaining the wound’s moisture while boosting epithelial cell migration, accelerating collagen maturation, and reducing inflammation [105]. After that, an increase in the amount of collagen, hyaluronic acid, and dermatan sulfate in the healing wound’s granulation tissue is observed, as is an increase in blood supply as a result of improved oxygenation [106]. 

The outcomes presented in Table 5 confirmed that *P. tenuifolia* petal extracts can stimulate keratinocyte growth and migration; the most efficient was the aqueous extract of the petals from Gulenovci obtained by the MAE method, followed by the petal extract from Deliblato sands acquired by the maceration method. Somewhat less effective was the UAE methanolic petal extract from Gulenovci (8.04% more efficient in comparison to the control), followed by the methanolic extracts of the petals from Pančevo (by MAE) and Gulenovci (by maceration), which had a similar influence on cell migration (5.56% and 5.38%, respectively). 

No one has ever before reported such results, and to the best of our knowledge, these are the first findings providing proof that *P. tenuifolia* petal extracts enhance keratinocyte migration and proliferation. It is well documented that keratinocyte migration and proliferation are needed for wound skin re-epithelialization [107] which is a crucial step for restoring the new epidermis for injured skin repair [108]. The aqueous MAE extract of the petals had the highest wound healing activity had the lowest content of quercetin and isorhamnetin (0.159 and 0.26 mg/L, respectively), whereas *p*-coumaric acid was not detected in it. The other extracts with the wound healing potential had varying concentrations of the quantified components (*p*-coumaric acid, ellagic acid, quercetin, and isorhamnetin), which led to the difference in their activities. Hence, our findings indicate that *P. tenuifolia* petals may aid in skin wound healing. To the best of the authors’ knowledge, extracts of the petals of no herbaceous peonies have been tested for their effects on wound healing on HaCaT cells in the past. 

### 3.5. Adhesion and Invasion Capacities to HaCaT Cells by S. lugdunensis

Bacterial pathogens can infiltrate and colonize the surface of host cells and tissues despite a wide range of resistance systems in cultured cells or tissues. The current study looked at the antibacterial activity of *P. tenuifolia* aqueous and methanolic extracts against *S. lugdunensis* in the infected HaCaT cell line. The cell invasion activity in HaCaT cells was evaluated using the extract’s subtoxic and minimal inhibitory concentrations. As a result, the ability to inhibit adhesion and invasion of *S. lugdunensis* on HaCaT cells was tested at 0.125 mg/mL (Figure 2). The highest inhibitory activity of the extracts could be associated with the presence of the lowest content of quercetin and isorhamnetin among all of the tested extracts, whereas *p*-coumaric acid was not detected in this sample. To the best of the authors’ knowledge, this is the first study conducted on the effects of the extracts of herbaceous peonies on *S. lugdunensis* adhesion and invasion of human keratinocytes. Because of the promising results, the examined extracts are possible contenders for generating innovative topical formulations to address skin infections. 

## 4. Materials and Methods

### 4.1. Origin of Plant Material

**Figure 4:**
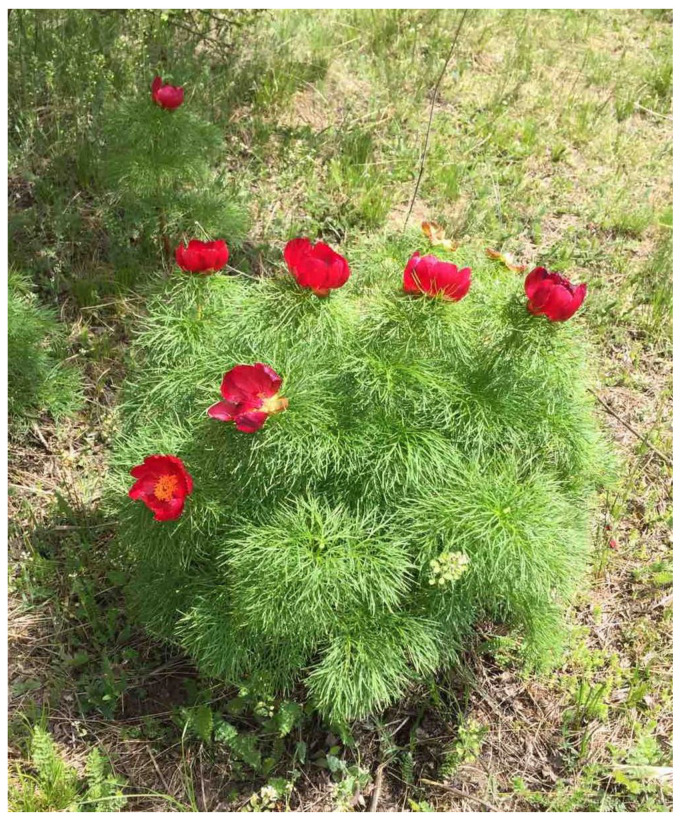
Full blooming *Paeonia tenuifolia* L., at locality Deliblato sands, Serbia (May 2022).

Fresh petals of *Paeonia tenuifolia* L. were collected in May 2022 from plants growing spontaneously in their natural habitats in Serbia, namely Deliblato sands at 167 m a.s.l. (8 May, Figure 4) and Gulenovci at 840 m a.s.l. (11 May), as well as from plants domesticated in the Institute’s collection in Pančevo, Serbia 74 m a.s.l. (12 May). Wild-collecting was conducted with the permission of the Ministry of Environmental Protection of the Republic of Serbia (No. 353-01-162/2022-04, issued on 24 February 2022). The collection of petals was performed manually from randomly selected full-blooming plants. At each locality, 1/3 of the petals per flower were collected from less than 10% of the flowering plants found. The collected petals were shade-dried at room temperature prior to being further subjected to various extraction methods.

Following collection, voucher specimens of this strictly protected plant species in Serbia were confirmed and deposited at the Herbarium BUNS at the Department of Biology and Ecology, Faculty of Sciences, University of Novi Sad, Serbia: (1) *Paeonia tenuifolia* L., Deliblato sands, Serbia, BUNS 2-2105; (2) *P. tenuifolia* L., Gulenovci, Serbia, BUNS 2-0686; (3) *P. tenuifolia* L., Pančevo, Serbia, BUNS 2-2106.

### 4.2. Extraction of Plant Material 

#### 4.2.1. Extraction by Maceration Method

The maceration method of extraction of the petals was carried out using a linear mechanical homogenizer (Roller mixer SRT6, Germany) at 25 ± 5 °C for 24 h with methyl alcohol as a solvent. When the solvent of choice was distilled water, the maceration was carried out at 80 °C for 15 min. A laboratory filter paper was used to filter the extracts obtained from the petals (5 g), using 100 mL of methyl alcohol (distilled water). Until further analysis, the collected analyte was kept in a dark bottle at 4 °C.

#### 4.2.2. Ultrasound-Assisted Extraction (UAE)

An ultrasonic processor of 750 W output with a 20 kHz converter and a 13 mm diameter solid titanium probe at a 70% amplitude was used to extract the ground petals (2.5 g) for 10 min at 60 °C (Cole-Parmer Ultrasonic Processor Stainless Steel Temperature Probe, UK). The same extraction procedure was performed using water as a solvent, while keeping the solid-to-solvent ratio at 1:20. The raw extracts were collected after the mixture had been filtered through laboratory filter paper and stored at 4 °C, awaiting further analysis.

#### 4.2.3. Microwave-Assisted Extraction (MAE)

Using a Microwave Synthesis Reactor (Monowave 300 from Anton Paar, GmbH in Germany, with the operating parameters presented in Figure S2), 10 mL of methyl alcohol was used in order to extract 0.5 g of the powdered petals at 60 °C for 10 min. The identical process was performed using distilled water as the solvent. The acquired petal extracts were filtered through a qualitative laboratory filter paper and kept in storage at 4 °C until further analysis.

### 4.3. Chemical Analysis

#### 4.3.1. Chemicals 

The following reagents were used: Folin–Ciocalteu reagent, 2,2-diphenyl-1-picrylhydrazyl (DPPH), potassium ferricyanide, gallic acid, catechin, Trolox, iron(II)sulfate, and iron(III)chloride were bought from Sigma Aldrich (USA), sodium-carbonate from Zdravlje (Serbia), sodium nitrite from Alkaloid Skopje (Macedonia), aluminum chloride and trichloroacetic acid were from Kemika (Croatia), sodium hydroxide from NRK Inzenjering (Serbia), 2,2’-azino-bis(3-ethylbenzothiazoline-6-sulfonic acid)-ABTS was from Roche Diagnostics GmbH (Germany), neocuproin was from Acros Organics (Belgium), monosodium phosphate and disodium phosphate were from Merck (USA), cuprum chloride was from Fluka (Germany), and ammonium acetate and ethanol were from Zorka Pharma (Serbia). 

#### 4.3.2. UHPLC-LTQ-Orbitrap MS

For LC/MS analysis (Thermo Fisher Scientific, Bremen, Germany) an LTQ OrbiTrap XL mass spectrometer linked to an Accela 600 UHPLC system running in positive and negative ionization mode (heated electrospray ionization, or HESI) was used. For separation, a Syncronis C18 analytical column (50 2.1 mm, 1.7 m particle size) was used. Prior reports on UHPLC conditions and MS parameters are provided in Zengin et al. [109]. The molecule editor program ChemDraw (version 12.0, CambridgeSoft, Cambridge, MA, USA) was used for structure drawing and for calculating the precise masses of the compounds of interest. 

Xcalibur software (ver. 2.1, Thermo Fisher Scientific, Waltham, MA, USA) was used for instrument control and data analysis. Some of the compounds for which no standards were available were tentatively identified using previously reported MS fragmentation data [110,111].

Chemical profiling of the methanolic extract of the petals was assessed by an advanced LC/MS method (UHPLC-LTQ-Orbitrap-MS). Deprotonated molecule mass [M-H]^−^ and MS^2^, MS^3^, and MS^4^ fragmentation behavior were used for the identification of compounds in the extract with the assistance of the available literature data.

#### 4.3.3. UHPLC/MS Target Analysis of Active Compounds 

A Dionex Ultimate 3000 UHPLC system with a TSQ Quantum Access Max triple-quadrupole mass spectrometer (ThermoFisher Scientific, Basel, Switzerland) was used to separate, identify, and quantify the components in the samples of *P. tenuifolia* petal extracts. On a Syncronis C18 column, the elution was carried out at 40 °C. Water + 0.1% formic acid (A) and acetonitrile (B) made up the mobile phase, which was administered in the following gradient elution: 5% B for the first 2.0 min, 5–95% B for the next 2.0–12.0 min, 95% B for the next 12.0–12.2 min, and 5% B for the last 15 min. The flow rate was set at 0.4 mL/min and the injection had a volume of 5 μL.

The temperature of the vaporizer was kept at 200 °C and the heated electrospray ionization (HESI) source was used with a TSQ Quantum Access Max triple quadrupole mass spectrometer with the following settings: spray voltage 5000 V, sheet gas (N_2_) pressure 40 AU, ion sweep gas pressure 1 AU, auxiliary gas (N_2_) pressure 8 AU, capillary temperature 300 °C, and skimmer offset 0 V. The mass spectrometry data were collected from 100 to 1000 *m*/*z* in negative ion mode. For the qualitative examination of the targeted chemicals, several mass spectrometric scanning modes, such as full scanning (FS) and product ion scanning (PIS), were used. The collision energy was adjusted depending on the compound in the collision-induced fragmentation tests, which were carried out with argon serving as the collision gas. The time-selected reaction monitoring (tSRM) studies for quantitative analysis were performed using two MS^2^ fragments for all the molecules previously identified as dominant in the PIS tests.

#### 4.3.4. Determination of the Content of Active Constituents in the Extracts

##### Total Polyphenol Content 

The total polyphenol content in the petal extracts was assessed using a modified Folin–Ciocalteu method described by Waterhouse [112]. Using the phenol reagent developed by Folin–Ciocalteu and spectrophotometric analysis, the total amount of phenols was assessed. In short, a 2000 μL flask was filled with 20 μL of adequately diluted extract, 100 μL of the Folic–Ciocalteu reagent, and 1500 μL of deionized water. After waiting for five minutes, 300 μL of sodium carbonate (20% *w*/*v*) was added, and the mixture was then filled up to 2000 μL with deionized water. Using a Shimadzu 1800 UV/Vis spectrophotometer (Japan), the absorbance at 765 nm was measured after 120 min of incubation in the dark at room temperature. The calibration curve was created using a gallic acid solution. Results are given in terms of milligrams of gallic acid equivalent per gram of dried plant material (mg GAE/g). Each analysis was run three times and the results were statistically processed.

##### Total Flavonoid Content 

Utilizing a modified technique described by Park et al. [113], the total flavonoid content (TFC) in the petal extracts was determined. In short, 1250 μL of deionized water was added to 250 μL of suitably diluted extract together with 75 μL of 5% sodium nitrite solution. The combination was then kept in the dark for five minutes. Following that, the mixture was treated with 150 μL of 10% Aluminum (III) chloride solution and 500 μL of 1M sodium hydroxide before being topped off with deionized water to a final volume of 2000 μL. At a wavelength of 425 nm, the samples’ absorbance was measured. A Shimadzu 1800 UV/Vis spectrophotometer (Japan) was used to perform each test in triplicate. The calibration curve was produced using catechin monohydrate. The results are given as mg of catechin equivalent per gram of dried plant material (mg CE/g).

#### 4.3.5. Antioxidant Assay 

As opposed to assays that quantify antioxidant activity as the percentage decrease in absorbance, the antioxidant activity in this study was expressed as mg of Trolox equivalents per gram of dried plant material for the CUPRAC assay, as a half-maximal inhibitory concentration (IC_50_) for ABTS and DPPH, whereas the FRAP assay is expressed as μmol Fe^2+^/g of plant material. This allows an easier and more direct comparison of the antioxidant activity. 

##### Ferric Reducing Antioxidant Power Assay

Reducing power activity quantifies the reductive ability that can be measured by converting a ferricyanide complex ([Fe(CN)_6_]^3−^) to ferrocyanide or ([Fe(CN)_6_]^4−^) via the action of an electron or hydrogen atom donated by an antioxidant [114]. The petal extract (10 mg) was mixed with 1 mL of the K_3_Fe(CN)_6_ solution and 1 mL of phosphate buffer (pH ≈ 6.6) and the mixture was incubated for 4 h at 50 °C. Following the incubation period, 0.25 mL of a 10% trichloroacetic acid solution is dissolved in 0.5 mL of the produced sample. Next, 0.75 mL of distilled water and 0.17 mL of FeCl_3_ (0.1% *m*/*v*) are added. All of the reagents—all but the extract—were present in the negative control. Three parallel runs of the experiment were conducted, and the absorbance was measured at 750 nm using the Shimadzu 1800 UV/Vis spectrophotometer (Japan). The results are given as μmol Fe^2+^/g of dried plant material and were calculated using ferrous sulfate to create the calibration curve.

##### Cupric Ion Reducing Antioxidant Capacity Assay

The mixture was made by mixing 0.8 mL of extract with 1 mL of CuCl_2_x2H_2_O (Copper(II) chloride dihydrate), 1 mL of neocuproine, and 1.2 mL of ammonium acetate buffer (pH ≈ 7). The sample was then incubated for 30 min at room temperature in complete darkness before the absorbance at 450 nm was determined using a Shimadzu 1800 UV/Vis spectrophotometer (Japan). For each extract, the assay result was verified three times. Trolox was used to obtain the calibration curve for this methodology. In terms of Trolox equivalent (TE), the obtained values are given as mol TE/g of dried plant material.

##### ABTS Assay

The analytical protocol described by Prior et al., with some modifications [59,115,116], served as the foundation for the ABTS^●+^ scavenging assay. Specifically, 200 μL of the petal extract and 2800 μL of the ABTS^•+^ radical cation solution was combined and incubated at 25 ± 5 °C in the dark for 30 min. The ABTS^•+^ radical solution (7.8 mmol/L) was made by dissolving 20 mg of ABTS(s) in 5 mL of deionized water and then adding 88 μL of potassium-persulfate (aq) solution with a concentration of 2.45 mmol/L (380 mg of potassium-persulfate(s) was dissolved in 10 mL of deionized water). Prior to use, the ABTS stock solution mixture was incubated for 16 to 20 h at 4 °C in the dark to create (activate) the radical cation solution (ABTS^•+^). Following activation, the ABTS^•+^ radical cation solution was diluted by methyl alcohol/distilled water, yielding an initial absorbance of 0.7 ± 0.02 at a wavelength of 734 nm. The control solution (blank) was made by mixing 2800 μL of ABTS^•+^ radical cation solution with 200 μL of extraction solvent in place of extract. A triple of each measurement was made. Using a Shimadzu 1800 UV Spectrophotometer (Japan), the absorbance of each sample was calculated at 734 nm. The radical scavenging activity was calculated according to the equation:

(1) $$SC_{ABTS}=\frac{A_{cont}-A_{sample}}{A_{cont}}\times 100\%$$

where ***A_cont_*** represents the absorbance value of the blank solution, while ***A_sample_*** is the absorbance of the extract sample treated with the ABTS^•+^ cation, and the results are presented as IC_50_ (mg/mL), the concentration of the extract required to neutralize 50% of ABTS^●+^ radicals.

##### DPPH Assay

The DPPH solution was created by combining 9 mL of ethyl alcohol with 0.252 mg of DPPH. The petal extract was then combined with 2.8 mL of this solution, which was then left at room temperature for 30 min, without any illumination. The test was run concurrently with other projects. The scavenging activity (***SC_DPPH_***) was calculated using the following equation after the absorbances were measured at 517 nm:

(2) $$SC_{DPPH}=\frac{A_{cont}-A_{sample}}{A_{cont}}\times 100\%\;$$

The calibration curve was created using Trolox solution, and the results are presented as IC_50_ (mg/mL), the concentration of the extract required to neutralize 50% of DPPH^●^ radicals.

#### 4.3.6. Antimicrobial and Antibiofilm Activities of Methanolic and Aqueous Extracts

##### Antibacterial Activity

The extract’s activity was tested on Gram-positive bacteria (*Staphylococcus aureus* ATCC 11632 and *Staphylococcus lugdunensis* Ibis 2996) and Gram-negative bacteria (*Proteus vulgaris* IBR P004). Following a process previously described [20], the microdilution method (96-well microtiter plates) was used to determine the minimum inhibitory concentration (MIC) and minimum bactericidal concentration (MBC). The extracts were diluted in 30% ethanol, added to a tryptic soy broth (TSB, Torlak, Serbia) medium, and then inoculated with bacteria at a final concentration of 1 × 10^6^ colony-forming units (CFU) per well. The positive control included gentamicin (Panfarma, Belgrade, Serbia). Values for the MIC and MBC were given in mg/mL.

##### Antifungal Activity

The extract’s activity was tested on the following *Candida* strains: *Candida albicans* (Y177), *Candida kefyr* (Y289), and *Candida krusei* (Y454). The anticandidal assay was conducted by the modified EUCAST procedure (EUCAST, 2002) as previously described [117]. Ketoconazole was used as a positive control.

##### Bacterial Biofilm Inhibitory Activity

The effects of six different extracts on the *S. lugdunensis* biofilm were assessed, with minor alterations, as previously described in Smiljkovic et al. [11]. In order to create a biofilm, *S. lugdunensis* was cultured in Triptic soy broth with 2% glucose in 96-well microtiter plates with adhesive bottoms (Sarstedt, Germany) with the MIC, MIC/2, and MIC/4 of extracts at 37 °C for 24 h. Following the incubation, the wells were twice rinsed with sterile PBS. After that, the biofilm was fixed with methyl alcohol, air-dried, and each well was rinsed twice with PBS. Crystal violet (Bio-Merieux, France) was used to stain the biofilm for 30 min. After incubation, the crystal violet had been eliminated, the wells had been cleaned with water, dried by air, and then 96% ethanol (Zorka, Serbia) was employed. Thermo Scientific’s Multiskan FC Microplate Photometer was used to measure absorbance at 620 nm and Equation (3) was used to determine the percentage of biofilm destruction.

(3) $$Biofilm\;destruction\;\left(\%\right)=\left[\frac{A_{620,\;control}-A_{620,\;sample}}{A_{620,\;control}}\right]\times 100\%$$

#### 4.3.7. Cytotoxicity of the Extracts

The extract’s activity was tested on the human keratinocyte (HaCaT) cell line, obtained from AddexBio (San Diego, CA, USA, catalogue number T0020001). By employing the crystal violet technique, previously described by Stojkovic et al. [118], the cytotoxic effect of petal extracts was assessed on a spontaneously immortalized keratinocyte cell line (HaCaT). The extracts were dissolved in PBS to a final concentration of 8 mg/mL. The HaCaT cells were grown in high-glucose Dulbecco’s Modified Eagle Medium (DMEM) supplemented with 10% fetal bovine serum (FBS), 2 mM L-glutamine, and 1% penicillin and streptomycin (Invitrogen) at 37 °C in a 5% CO_2_ incubator. Cells were seeded in a 96-well microtiter adhesive plate for 48 h. After that period, the medium was withdrawn, and the cells were treated for the following 24 h in duplicate wells with varying concentrations of the extract. After removing the medium, the cells were given two PBS washes before being stained for 20 min at room temperature with a 0.4% crystal violet staining solution. The cells were then washed in a stream of tap water, by which the crystal violet staining solution was removed, and left to air dry at room temperature. The absorbance was measured at 570 nm (OD570) in a plate reader. The results were expressed as an IC_50_ value, indicating 50% growth inhibition of HaCaT cells when compared to an untreated control.

#### 4.3.8. Scratch Wound Healing Assay

With a few adjustments, the test was carried out as described in Stojkovic et al. [118]. Confluent HaCaT cells were produced. A 200 μL tip was used to scrape the cell monolayer. After washing the floating cells, the cells were cultured in DMEM containing 250 g/mL of IC_25_ doses of the extracts. This DMEM was supplemented with 1% FBS, 2 mM L-glutamine, and 1% antibiotic-antimycotic (Invitrogen), and 24 h after the wound was formed, cell migration was examined using a Nikon Eclipse TS2 microscope (Amsterdam, Netherlands). Cells that were not treated served as a control. Percentages of wound closure during the exposure to the extracts were used to display the results. There were three separate experiments conducted.

#### 4.3.9. Effects of the on the Adhesion and Invasion Capacities of *S. lugdunensis* to HaCaT Cells

As the extracts of petals collected at the locality Gulenovci revealed the lowest MIC values in the antimicrobial activity assay, they were selected to further examine the effects of the adhesion and invasion capacities of *S. lugdunensis* on HaCaT cells. With some modifications of the method described in Ahmed et al. [119], the potential of selected extracts to lessen *S. lugdunensis*’s capacity to adhere to and invade HaCaT cells was assessed. 

HaCaT cells were expanded to confluence in 24-well plates with adhesive bottoms. Following the removal of the medium, the cells were then given fresh DMEM without the FBS containing the extract. In order to assess the ability of the cells to adhere, 100 L of *S. lugdunensis* culture (10^8^ CFU/mL) was introduced to the cells after 15 min of incubation at 37 °C. In order to examine the invasion capacity of the bacteria, HaCaT cells were cultured with the bacteria for 2 h at 37 °C. The adhering bacteria were then killed by treating the HaCaT cells with gentamicin (300 g/mL) for 1 h. The cells were then lysed with 1 mL of 1% (*v*/*v*) Tween-20 (Sigma Aldrich, Darmstadt, Germany) at 37 °C for 30 min. Following three rounds of washing with DMEM without FBS, the cells were then used. After that, Trypton Soy Agar plates were seeded with diluted versions of the *S. lugdunensis* suspension from each well. After 18 h of incubation at 37 °C, the number of CFU was calculated.

### 4.4. Statistical Analysis 

The statistical analysis was performed using one-way analysis of variance (one-way ANOVA) and Duncan’s post hoc test using the software STATISTICA 7.0. The differences were deemed statistically significant at *p* < 0.05, n = 3, and the sample size was three.

## 5. Conclusions

The aim of this study was to determine the chemical composition of the aqueous and methanolic extracts of *P. tenuifolia* petals originating from different localities in Serbia, as well as to estimate their skin-beneficial biological activities. In short, when water is used as an extraction solvent for the petals of *P. tenuifolia*, the MAE method proved to be the most favorable extraction procedure used to produce extracts with the most beneficial skin-related therapeutic activities, followed by the UAE method. As the research showed, the activity of the extracts varied between the localities from which the petals were collected. In order to obtain a standardized herbal material, further research should be focused on the detailed determination of chemical compounds in all of the extracts, which would provide information about the locality from which the petals should be collected, or *P. tenuifolia* further cultivated, for possible use in the pharmaceutical and cosmetic industries.

## Figures and Tables

**Table 1:** Total polyphenol (TPC) and total flavonoid content (TFC) of *Paeonia tenuifolia* L. extracts obtained by different extraction methods and extraction solvents.

Origin of Plant Material	Extraction Medium	Extraction Method	TPC [mg GAE/g]	TFC [mg CE/g]
Gulenovci	H_2_O	Maceration	31.34 ± 0.23 ^e^	24.62 ± 0.11 ^b^
		UAE	22.82 ± 0.21 ^c^	15.75 ± 0.20 ^f^
		MAE	28.22 ± 0.27 ^a^	23.09 ± 0.21 ^a^
	MeOH	Maceration	31.85 ± 0.31 ^e^	24.50 ± 0.19 ^c^
		UAE	32.14 ± 0.22 ^d^	20.23 ± 0.25 ^e^
		MAE	35.24 ± 0.23 ^b^	21.44 ± 0.11 ^d^
Pančevo	H_2_O	Maceration	24.04 ± 0.32 ^c^	16.54 ± 0.23 ^f^
		UAE	18.91 ± 0.47 ^e^	12.31 ± 0.19 ^e^
		MAE	22.51 ± 0.25 ^d^	16.78 ± 0.25 ^d^
	MeOH	Maceration	18.43 ± 0.24 ^e^	12.69 ± 0.18 ^f^
		UAE	22.08 ± 0.26 ^d^	15.78 ± 0.27 ^f^
		MAE	28.15 ± 0.33 ^b^	19.73 ± 0.23 ^c^
Deliblato sand	H_2_O	Maceration	30.15 ± 0.33 ^d^	24.61 ± 0.32 ^d^
		UAE	32.94 ± 0.26 ^e^	25.44 ± 0.37 ^c^
		MAE	33.26 ± 0.15 ^e^	28.48 ± 0.18 ^d^
	MeOH	Maceration	23.54 ± 0.20 ^d^	16.01 ± 0.29 ^e,f^
		UAE	32.83 ± 0.19 ^e^	23.72 ± 0.44 ^e^
		MAE	26.04 ± 0.26 ^c^	20.84 ± 0.41 ^f^

Values with different letters (a–f) in each column showed statistically significant differences (*p* < 0.05; n = 3; analysis of variance, Duncan’s post-hoc test); Ultrasound-assisted extraction (UAE); Microwave-assisted extraction (MAE), Methyl alcohol (MeOH), Gallic acid equivalent (GAE); Catechin equivalent (CE); mg of gallic acid equivalent per g of dried plant material (mg GAE/g); mg of catechin equivalent per g of dried plant material (mg CE/g).

**Table 2:** Antioxidant (DPPH, ABTS, CUPRAC, and FRAP) activity of water and methyl alcohol extracts of the petals of *Paeonia tenuifolia* L.

Origin of Plant Material	Extraction Medium, Extraction Method	Antioxidant Assays			
		DPPH IC_50_ [mg/mL]	ABTS IC_50_ [mg/mL]	CUPRAC [mol TE/g]	FRAP [μmol Fe^2+^/g]
Gulenovci	H_2_O, maceration	0.088 ± 0.001 ^c^	0.090 ± 0.001 ^b^	0.386 ± 0.002 ^a^	834.24 ± 6.4 ^e^
	H_2_O, UAE	0.051 ± 0.001 ^a^	0.074 ± 0.002 ^a^	0.378 ± 0.001 ^b^	843.39 ± 5.6 ^e^
	H_2_O, MAE	0.063 ± 0.001 ^b^	0.092 ± 0.001 ^b^	0.327 ± 0.001 ^f^	796.56 ± 10.5 ^c^
	MeOH, maceration	0.123 ± 0.001 ^d^	0.099 ± 0.000 ^c^	0.345 ± 0.000 ^e^	830.22 ± 11.6 ^e^
	MeOH, UAE	0.124 ± 0.001 ^d^	0.099 ± 0.001 ^c^	0.349 ± 0.001 ^d^	776.43 ± 9.9 ^d^
	MeOH, MAE	0.124 ± 0.002 ^d^	0.098 ± 0.001 ^c^	0.358 ± 0.002 ^b^	840.46 ± 7.0 ^e^
Pančevo	H_2_O, maceration	0.074 ± 0.002 ^b^	0.089 ± 0.001 ^b^	0.341 ± 0.001 ^f^	715.33 ± 5.8 ^e^
	H_2_O, UAE	0.058 ± 0.001 ^a^	0.070 ± 0.002 ^a^	0.371 ± 0.001 ^b^	832.4 ± 13.9 ^b^
	H_2_O, MAE	0.061 ± 0.001 ^c^	0.088 ± 0.002 ^b^	0.385 ± 0.002 ^a^	751.19 ± 5.4 ^d^
	MeOH, maceration	0.126 ± 0.001 ^d^	0.097 ± 0.001 ^c^	0.346 ± 0.001 ^e^	724.84 ± 10.3 ^e^
	MeOH, UAE	0.126 ± 0.001 ^d^	0.099 ± 0.000 ^c^	0.357 ± 0.001 ^d^	777.16 ± 9.8 ^c^
	MeOH, MAE	0.125 ± 0.002 ^d^	0.099 ± 0.000 ^c^	0.367 ± 0.001 ^c^	748.99 ± 8.2 ^d^
Deliblato sands	H_2_O, maceration	0.103 ± 0.003 ^c^	0.098 ± 0.001 ^c^	0.319 ± 0.001 ^e^	759.24 ± 2.3 ^e^
	H_2_O, UAE	0.094 ± 0.003 ^b^	0.092 ± 0.001 ^b^	0.344 ± 0.001 ^c^	815.58 ± 1.9 ^b^
	H_2_O, MAE	0.067 ± 0.001 ^a^	0.094 ± 0.001 ^a,b^	0.391 ± 0.000 ^b^	791.43 ± 7.8 ^c^
	MeOH, maceration	0.125 ± 0.001 ^d^	0.098 ± 0.001 ^c^	0.353 ± 0.001 ^f^	769.11 ± 10.8 ^e^
	MeOH, UAE	0.125 ± 0.001 ^d^	0.097 ± 0.001 ^c^	0.337 ± 0.001 ^d^	559.5 ± 11.3 ^d^
	MeOH, MAE	0.125 ± 0.002 ^d^	0.097 ± 0.001 ^c^	0.358 ± 0.001 ^f^	833.88 ± 8.6 ^a^

Values with different letters (a–f) in each column showed statistically significant differences (*p* < 0.05; n = 3; analysis of variance, Duncan’s post-hoc test); Ultrasound-assisted extraction (UAE); Microwave-assisted extraction (MAE); Methyl alcohol (MeOH); Trolox equivalent (TE); the concentration of the extract required to neutralize 50% of DPPH^●^ and ABTS^●+^ radicals (IC_50_).

**Table 3:** Antibacterial activity of *Paeonia tenuifolia* L. water and methanolic extracts (MIC and MBC, mg/mL).

Origin of Plant Material	Extraction Medium, Extraction Method	Bacteria					
		*S. lugdunensis*		*S. aureus*		*P. vulgaris*	
		MIC	MBC	MIC	MBC	MIC	MBC
Gulenovci	H_2_O, maceration	0.125	0.25	0.5	1	0.5	1
	H_2_O, UAE	0.5	1	2	4	2	4
	H_2_O, MAE	0.5	1	2	4	0.125	0.25
	MeOH, maceration	0.25	0.5	0.25	0.5	0.25	0.5
	MeOH, UAE	0.25	0.5	0.5	1	0.25	0.5
	MeOH, MAE	0.5	1	1	2	0.5	1
Pančevo	H_2_O, maceration	0.5	1	4	8	2	4
	H_2_O, UAE	1	2	0.25	0.5	2	4
	H_2_O, MAE	0.5	1	2	4	1	2
	MeOH, maceration	0.5	1	1	2	1	2
	MeOH, UAE	0.5	1	0.5	1	0.5	1
	MeOH, MAE	0.5	1	1	2	0.5	1
Deliblato sands	H_2_O, maceration	0.5	1	2	4	2	4
	H_2_O, UAE	0.5	1	1	2	1	2
	H_2_O, MAE	0.5	1	2	4	2	4
	MeOH, maceration	1	2	2	4	1	2
	MeOH, UAE	1	2	0.5	1	0.5	1
	MeOH, MAE	0.5	1	0.5	1	0.5	1
Control	Gentamicin	0.008	0.016	1.33	2.66	0.066	0.133

Minimal inhibitory concentration (MIC); Minimal bactericidal concentration (MBC); Ultrasound-assisted extraction (UAE); Microwave-assisted extraction (MAE), Methyl alcohol (MeOH).

**Table 4:** Anticandidal activity of *Paeonia tenuifolia* water and methanolic extracts (MIC and MFC, mg/mL).

Origin of Plant Material	Extraction Medium, Extraction Method	*Candida* Species					
		*C. kefyr*			*C. krusei*		*C. albicans*
		MIC	MFC	MIC	MFC	MIC	MFC
Gulenovci	H_2_O, maceration	1	2	0.5	1	1	2
	H_2_O, UAE	0.5	1	2	4	1	2
	H_2_O, MAE	0.5	1	1	2	1	2
	MeOH, maceration	1	2	0.5	1	1	2
	MeOH, UAE	0.5	1	1	2	1	2
	MeOH, MAE	0.5	1	1	2	0.5	1
Pančevo	H_2_O, maceration	0.5	1	2	4	1	2
	H_2_O, UAE	1	2	2	4	1	2
	H_2_O, MAE	0.5	1	1	2	1	2
	MeOH, maceration	0.5	1	1	2	0.5	1
	MeOH, UAE	0.5	1	1	2	1	2
	MeOH, MAE	1	2	1	2	0.5	1
Deliblato sands	H_2_O, maceration	0.5	1	2	4	1	2
	H_2_O, UAE	1	2	1	2	1	2
	H_2_O, MAE	0.5	1	2	4	1	2
	MeOH, maceration	1	2	0.5	1	1	2
	MeOH, UAE	1	2	1	2	1	2
	MeOH, MAE	0.5	1	1	2	0.5	1
Control	Ketoconazole	0.05	0.1	0.05	0.1	0.05	0.1

Ultrasound-assisted extraction (UAE); Microwave-assisted extraction (MAE); Methyl alcohol (MeOH); Minimal inhibitory concentration (MIC); Minimal fungicidal concentration (MFC).

**Table 5:** Effects of the *P. tenuifolia* extracts on migration capacity of HaCaT cells (wound healing).

Origin of Plant Material	Extraction Medium	Extraction Method	Wound Healing (%)
Gulenovci	H_2_O	MAE	26.14 ± 0.04
	MeOH	Maceratiom	5.38 ± 1.2
		UAE	8.04 ± 0.11
		MAE	0.08 ± 0.7
Pančevo	MeOH	UAE	NA
		MAE	5.56 ± 0.09
Deliblato sands	MeOH	Maceration	19.19 ± 1.3
		MAE	NA
Control			0.08 ± 0.12

No activity (NA); Ultrasound-assisted extraction (UAE); Microwave-assisted extraction (MAE); Methyl alcohol (MeOH).

## Data Availability

Data is contained within the article and Supplementary Materials.

## Supplementary Materials

The following supporting information can be downloaded at: https://www.mdpi.com/article/10.3390/ph15121537/s1, Table S1: HRMS and MS^4^ data for metabolites identified in *P. tenuifolia* methanolic extract. Table S2: Quantification of active compounds in the extracts of *P. tenuifolia*, Figure S1: Trolox calibration curve for DPPH^●^ radical scavenging activity, Figure S2: Energy input (blue line—power [W]), pressure (green line—in bars) and temperature (yellow line—temp IR [°C]).
