# Supplementary material for: Chemical Profile and Skin-Beneficial Activities of the Petal Extracts of Paeonia tenuifolia L. from Serbia
Source: Pharmaceuticals (Basel). 2022 Dec 11;15(12):1537. doi: 10.3390/ph15121537 (PMC9787298; doi:10.3390/ph15121537)
Supplement: Supplementary file 1 [file pharmaceuticals-15-01537-s001.zip › Figure S2.pdf]

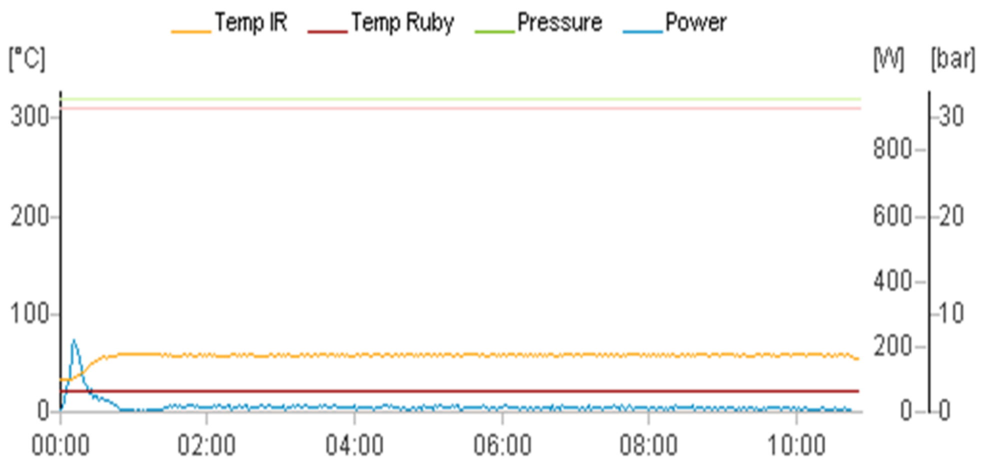

Figure S2 Energy input (blue line – power [W]), pressure (green line – in bars) and temperature (yellow line – temp IR [°C])
