# Supplementary material for: Chemical Profile and Skin-Beneficial Activities of the Petal Extracts of Paeonia tenuifolia L. from Serbia
Source: Pharmaceuticals (Basel). 2022 Dec 11;15(12):1537. doi: 10.3390/ph15121537 (PMC9787298; doi:10.3390/ph15121537)
Supplement: Supplementary file 1 [file pharmaceuticals-15-01537-s001.zip › Table S1.pdf]

Table S1 HRMS and MS^4 data for metabolites identified in *P. tenuifolia* methanolic extract.

No	Compound name	t_R, min	Molecular formula, $[M–H]^-$ /$[M+H]^+$	Calculated mass, $[M–H]^-$ /$[M+H]^+$	Exact mass, $[M–H]^-$ /$[M+H]^+$	Δ ppm	MS^2 Fragments, (% Base Peak)	MS^3 Fragments, (% Base Peak)	MS^4 Fragments, (% Base Peak)
Phenolic acids									
1	**Galloyl-hexoside 1**	0.57	$C_{13}H_{15}O_{10}^-$	331.06707	331.06580	3.82	125(8), 151(4), **169**(100), 170(4), 193(11), 211(21), 271(42)	**125**(100)	68(15), 81(63), 97(48), **107**(100)
2	**Galloyl-hexoside 2**	1.22	$C_{13}H_{15}O_{10}^-$	331.06707	331.06622	2.56	125(7), **169**(100), 170(4), 193(6), 211(17), 271(41), 272(3)	**125**(100)	69(10), 81(6), 97(35), **107**(100)
3	**Gallic acid**	1.28	$C_7H_5O_5^-$	169.01425	169.01426	-0.09	**125**(100)	**81**(100), 97(9)	
4	**Galloyl-hexoside 3**	1.78	$C_{13}H_{15}O_{10}^-$	331.06707	331.06629	2.35	168(8), 169(26), 211(5), 241(10), **271**(100), 272(11), 313(8)	169(13), **211**(100)	124(27), 125(7), 139(3), 165(11), 167(30), **168**(100), 183(9)
5	**Galloyl-hexoside 4**	2.13	$C_{13}H_{15}O_{10}^-$	331.06707	331.06612	2.86	125(8), **169**(100), 170(6), 211(3), 241(8), 271(5), 313(6)	**125**(100)	81(36), 97(55), **107**(100)
6	**Dihydroxybenzoyl-hexoside 1**	2.52	$C_{13}H_{15}O_9^-$	315.07216	315.07134	2.59	108(11), 109(13), 152(50), **153**(100), 163(8), 165(14), 278(14)	**109**(100)	
7	**Dihydroxybenzoic acid 1**	2.56	$C_7H_5O_4^-$	153.01933	153.01930	0.21	**109**(100), 110(5)	65(36), 67(13), **81**(100)	
8	**Galloyl-di-*O*-hexoside 1**	2.58	$C_{19}H_{25}O_{15}^-$	493.11989	493.11896	1.89	169(18), 241(16), 271(16), 283(22), **313**(100), 314(13), 331(28)	125(22), 151(8), **169**(100), 223(32), 241(10), 283(77), 295(12)	**125**(100)
9	**Galloyl-hexoside 5**	3.17	$C_{13}H_{15}O_{10}^-$	331.06707	331.06646	1.85	125(5), **169**(100), 170(6)	**125**(100)	81(68), 97(47), **107**(100)
10	**Galloyl-shikimic acid**	3.22	$C_{14}H_{13}O_9^-$	325.05651	325.05591	1.84	125(13), 164(4), **169**(100), 170(8), 236(17), 278(9), 281(6)	**125**(100)	**81**(100), 97(18)
11	**Dihydroxybenzoyl-hexoside 2**	3.26	$C_{13}H_{15}O_9^-$	315.07216	315.07151	2.06	109(11), 147(13), 152(44), **153**(100), 165(12), 191(87), 295(12)	**109**(100)	**81**(100)
12	**Galloyl-di-*O*-hexoside 2**	3.29	$C_{19}H_{25}O_{15}^-$	493.11989	493.11934	1.13	169(13), 271(8), **313**(100), 314(15), 331(4)	125(24), 151(18), **169**(100), 211(16), 223(39), 241(20), 295(14)	**125**(100)
13	**Digalloyl-hexoside 1**	3.29	$C_{20}H_{19}O_{14}^-$	483.07803	483.07733	1.45	**169**(100), 170(6), 193(5), 271(7), 313(16), 331(31), 332(4)	**125**(100)	79(16), **81**(100), 107(7)
14	**Hydroxybenzoyl-hexoside**	3.43	$C_{13}H_{15}O_8^-$	299.07724	299.07690	1.14	**137**(100), 169(14), 179(61), 209(32), 239(97), 240(10), 253(21)	**93**(100)	
15	**Digalloyl-hexoside 2**	3.49	$C_{20}H_{19}O_{14}^-$	483.07803	483.07729	1.52	**169**(100), 170(6), 193(6), 211(3), 271(6), 313(14), 331(17)	**125**(100)	69(76), 79(7), **81**(100), 83(5), 97(12), 125(6)
16	**Gallic acid rhamnoside**	3.62	$C_{13}H_{15}O_9$–	315.07216	315.07184	1.02	125(14), 151(18), 153(43), **169**(100), 191(12), 211(25), 255(9)	**125**(100)	**107**(100)
17	**1,7-di-*O*-Galloyl-sedoheptulose**	3.67	$C_{21}H_{21}O_{15}^-$	513.08859	513.08866	-0.13	329(6), **331**(100), 332(11)	125(11), 151(6), **169**(100), 193(14), 211(37), 241(4), 271(82)	**125**(100)
18	**Methyl galloyl-hexoside**	3.67	$C_{14}H_{17}O_{10}^-$	345.08272	345.08244	0.80	124(21), 125(38), 151(38), **169**(100), 183(40), 281(20), 313(48)	107(3), **125**(100)	**53**(100), 107(34)
19	**Digallic acid 1**	3.70	$C_{14}H_9O_9^-$	321.02521	321.02490	0.94	125(3), **169**(100), 170(7)	**125**(100)	69(75), 81(50), **97**(100), 125(15)
20	**Vanniloyl-hexoside (Mudanoside A)**	3.76	$C_{14}H_{17}O_9^-$	329.08781	329.08752	0.87	149(19), **167**(100), 169(20), 209(83), 211(14), 239(16), 269(25)	108(10), **123**(100), 149(26), 152(40)	**108**(100)
21	**Digalloyl-hexoside 3**	4.02	$C_{20}H_{19}O_{14}^-$	483.07803	483.07735	1.40	169(6), 193(7), 211(8), **271**(100), 272(6), 313(13), 331(13)	169(8), **211**(100)	124(18), 125(6), 139(5), 165(9), 167(30), **168**(100), 183(6)

**22**	**Galloyl-norbergenin**	4.03	$C_{20}H_{19}O_{13}^{+}$	467.08202	467.08083	2.54	**153**(100), 154(5), 237(13), 261(5), 279(10), 297(19), 449(16)	79(4), **125**(100), 143(27)	79(59), **97**(100), 107(14)
**23**	**Digallic acid 2**	4.07	$C_{14}H_{9}O_{9}^{-}$	321.02521	321.02503	0.55	**169**(100), 170(4)	**125**(100)	69(46), **81**(100), 97(18), 107(16)
**24**	**HHDP-hexoside**	4.44	$C_{21}H_{21}O_{13}^{-}$	481.09877	481.10014	-2.87	165(4), **301**(100), 319(14)	**165**(100)	97(7), 121(21), **137**(100)
**25**	**Dihydroxybenzoic acid 2**	4.44	$C_{7}H_{5}O_{4}^{-}$	153.01933	153.01954	-1.37	97(8), 107(11), 108(13), **109**(100), 110(14), 111(7), 125(11)	65(50), **81**(100)	
**26**	**Tetragalloyl-hexoside 1**	4.82	$C_{34}H_{27}O_{22}^{-}$	787.09995	787.09964	0.39	465(4), 617(16), 618(4), **635**(100), 636(13)	271(14), 313(11), 405(7), 423(63), **465**(100), 483(67), 617(23)	169(30), 193(9), 235(15), 271(4), 295(16), **313**(100), 421(15)
**27**	**Trigalloyl-hexoside**	4.83	$C_{27}H_{23}O_{18}^{-}$	635.08899	635.08869	0.47	465(17), 466(4), **483**(100), 484(12)	169(8), 193(6), 211(10), **271**(100), 313(21), 331(20), 465(3)	169(13), **211**(100)
**28**	**Tetragalloyl-hexoside 2**	4.95	$C_{34}H_{27}O_{22}^{-}$	787.09995	787.09896	1.25	**617**(100), 618(15), 635(14), 636(3)	277(12), 295(21), 313(7), 447(31), 449(6), **465**(100), 573(6)	169(20), 193(6), 235(5), 247(4), 271(9), 295(17), **313**(100)
**29**	**Ellagic acid**	5.18	$C_{14}H_{5}O_{8}^{-}$	300.99899	300.99872	0.90	157(3), **185**(100), 201(15), 213(18), 229(90), 257(6)	133(7), 141(14), 147(14), 157(31), **157**(100), 175(8), 185(21)	
**30**	**Digallic acid methyl ester 1**	5.19	$C_{15}H_{11}O_{9}^{-}$	335.04086	335.04000	2.56	**183**(100), 184(5)	124(88), **168**(100), 183(4)	**124**(100)
**31**	**Tetragalloyl-hexoside 3**	5.22	$C_{34}H_{27}O_{22}^{-}$	787.09995	787.09845	1.90	465(4), 574(3), **617**(100), 618(15), 619(4), 635(12), 636(3)	211(11), 295(10), 403(59), 421(17), 447(64), **465**(100), 573(73)	169(24), 207(3), 235(15), 295(11), **313**(100), 421(3), 447(11)
**32**	**Digalloyl-HHDP-protoquercitol**	5.60	$C_{34}H_{27}O_{21}^{+}$	771.10394	771.10324	0.90	233(22), 237(20), 261(79), 279(48), **305**(100), 431(72), 601(42)	**153**(100)	79(4), **125**(100), 143(27)
**33**	**Digallic acid methyl ester 2**	5.74	$C_{15}H_{11}O_{9}^{-}$	335.04086	335.04023	1.87	**183**(100), 184(5)	124(86), **168**(100), 183(3)	**124**(100)
**34**	**Trigalloyl-HHDP-protoquercitol**	5.87	$C_{41}H_{31}O_{25}^{+}$	923.11489	923.11456	0.36	**305**(100), 413(41), 431(59), 456(18), 457(74), 583(43), 771(40)	**153**(100)	79(4), **125**(100), 143(19)
**35**	**Pentagalloyl-hexoside**	5.80	$C_{41}H_{31}O_{26}^{-}$	939.11091	939.10895	2.08	617(5), 769(50), 770(33), 771(3), **787**(100), 788(19)	403(3), 447(3), 465(7), 573(5), **617**(100), 635(17)	235(13), 295(14), 403(46), 421(18), 447(61), **465**(100), 573(75)
**36**	***p*-Coumaric acid**	6.30	$C_{9}H_{7}O_{3}^{-}$	163.04007	163.04024	-1.04	**119**(100)	**91**(100)	
**37**	**Hydroxybenzoyl-galloyl-hexoside**	6.44	$C_{20}H_{19}O_{12}^{-}$	451.08820	451.08875	-1.22	137(6), 169(6), 227(4), **313**(100), 314(10), 331(89), 332(12)	125(22), 137(52), 151(16), 168(58), **169**(100), 269(37), 295(15)	125(42), **151**(100)
**38**	**Ferulic acid**	6.50	$C_{10}H_{9}O_{4}^{-}$	193.05063	193.05064	-0.03	134(3), 169(33), **193**(100), 259(3)	134(74), **149**(100), 178(65)	134(100)
**39**	**Methyl gallate**	7.43	$C_{8}H_{7}O_{5}^{-}$	183.02990	183.02900	4.92	124(96), 125(5), 153(44), 167(7), **168**(100), 169(6), 183(10)	**124**(100)	54(12), 78(100)
Flavonoid glycosides and aglycones									
**40**	**Taxifolin 3,7-di-*O*-hexoside**	3.77	$C_{27}H_{31}O_{17}^{-}$	627.15667	627.15641	0.42	267(7), 285(13), 303(10), 355(8), 447(18), **465**(100), 466(11)	167(3), 241(14), **285**(100), 303(59), 329(3), 339(9)	149(13), 199(12), 217(23), **241**(100), 242(7), 243(28), 257(8)
**41**	**Kaempferol 3,7-di-*O*-hexoside**	3.77	$C_{27}H_{29}O_{16}^{-}$	609.14611	609.14654	-0.70	285(7), **447**(100), 448(27)	284(5), **285**(100), 309(3)	125(49), 163(57), 199(28), 213(29), 217(53), 241(70), **257**(100)
**42**	**Chrysoeriol 7-*O*-hexoside**	4.08	$C_{22}H_{21}O_{11}^{-}$	461.10894	461.10849	0.96	293(4), 297(3), 298(5), **299**(100), 300(22), 301(7), 415(3)	147(8), 240(3), 256(7), 271(6), 283(20), **284**(100)	147(13), 214(10), 239(11), 240(21), **255**(100), 256(62), 267(11)
**43**	**Quercetin 3,7-di-*O*-hexoside**	4.24	$C_{27}H_{29}O_{17}^{-}$	625.14102	625.14160	-0.93	301(21), 302(3), 462(10), **463**(100), 464(11), 505(3)	271(3), 300(37), **301**(100), 343(7)	107(9), **151**(100), 179(70), 229(7), 255(13), 273(10), 301(16)
**44**	**Luteolin 7-*O*-hexoside**	4.30	$C_{21}H_{19}O_{11}^{-}$	447.09329	447.09333	-0.09	284(18), **285**(100), 286(12), 299(4), 321(3), 327(3), 429(3)	125(46), 163(35), 213(29), 217(55), **241**(100), 243(53), 257(98)	185(11), 197(47), 198(24), 199(28), **213**(100), 223(14), 241(13)

45	**Quercetin 3-*O*-hexoside-7-*O*-pentoside**	4.31	$C_{26}H_{27}O_{16}^{-}$	595.13046	595.13068	-0.38	301(34), 302(7), **433**(100), 434(20), 462(74), 463(72), 464(17)	179(3), 271(7), **300**(100), 301(35), 343(10), 344(3)	121(3), 151(8), 227(11), 254(12), 255(15), **271**(100), 272(12)
46	**Kaempferol 3-*O*-(2"-hexosyl)-hexoside**	4.54	$C_{27}H_{29}O_{16}^{-}$	609.14611	609.14635	-0.40	285(14), **447**(100), 448(11), 489(10)	151(4), 227(3), 255(15), 256(4), **284**(100), 285(48), 327(19)	227(14), **255**(100), 256(19)
47	**Quercetin 3-*O*-hexoside-7-*O*-rhamnoside**	5.00	$C_{27}H_{29}O_{16}^{-}$	609.14611	609.14627	-0.26	301(31), 302(4)), **447**(100), 448(11), 463(59), 464(6)	**301**(100)	107(11), **151**(100), 179(62), 229(14), 255(13), 273(9), 301(13)
48	**Kaempferol 3-*O*-pentoside-7-*O*-hexoside**	5.07	$C_{26}H_{27}O_{15}^{-}$	579.13554	579.13625	-1.22	263(30), 301(27), 407(72), 433(87), 446(85), **447**(100), 448(18)	299(9), **300**(16), 301(100)	**151**(100), 179(62), 211(14), 229(30), 255(38), 257(12), 301(14)
49	**Kaempferol 3-*O*-hexoside-7-*O*-rhamnoside**	5.26	$C_{27}H_{29}O_{15}^{-}$	593.15119	593.15042	1.31	285(16), 286(3), 431(37), 432(6), **447**(100), 448(10)	151(3), 227(3), 255(16), 256(3), **284**(100), 285(29), 327(19)	227(12), **255**(100), 256(23)
50	**Isorhamnetin 3-*O*-hexoside-7-*O*-rhamnoside**	5.34	$C_{28}H_{31}O_{16}^{-}$	623.16176	623.16160	0.25	315(7), 461(28), 462(4), **477**(100), 478(11)	271(5), 285(7), 299(5), **314**(100), 315(21), 329(3), 357(18)	243(28), 257(13), 271(81), **285**(100), 286(46), 299(12), 300(17)
51	**Quercetin 3-*O*-hexoside**	5.44	$C_{21}H_{19}O_{12}^{-}$	463.08820	463.08826	-0.13	300(38), **301**(100), 302(12)	151(81), **179**(100), 193(6), 229(6), 257(12), 272(8), 273(16)	**151**(100)
52	**Quercetin 3-*O*-pentoside**	5.62	$C_{20}H_{17}O_{11}^{-}$	433.07764	433.07732	0.74	**300**(100), 301(94), 302(10), 315(3), 343(5)	151(6), 179(8), 254(4), 255(53), 256(4), **271**(100), 272(12)	199(24), 203(9), 215(22), 227(60), 242(8), **243**(100), 271(32)
53	**Kaempferol 3-*O*-hexoside**	5.77	$C_{21}H_{19}O_{11}^{-}$	447.09329	447.09258	1.57	227(4), 255(19), 256(6), **284**(100), 285(95), 286(15), 327(17)	227(13), **255**(100), 256(21)	167(3), 183(3), 211(66), 213(3), **227**(100), 255(37)
54	**6-Hydroxykaempferol**	5.98	$C_{15}H_{9}O_{7}^{-}$	301.03538	301.03517	0.69	**257**(100), 258(9), 283(3)	147(55), 171(46), 189(69), 211(30), 213(59), 215(54), **229**(100)	159(9), 185(25), 187(20), **201**(100), 211(49), 214(8), 229(8)
55	**Quercetin**	6.80	$C_{15}H_{9}O_{7}^{-}$	301.03538	301.03521	0.55	107(5), 151(79), 152(5), **179**(100), 180(8), 257(12), 273(14)	**151**(100)	63(4), 65(3), 83(13), **107**(100)
56	**Onopordin**	7.41	$C_{16}H_{11}O_{7}^{-}$	315.05103	315.05087	0.49	**283**(100), 284(11)	195(3), 211(12), 215(7), 227(4), 239(15), **255**(100), 265(3)	183(16), 187(4), 209(3), 211(64), 213(13), **227**(100), 237(21)
57	**Limocitrin**	7.68	$C_{17}H_{13}O_{8}^{-}$	345.06159	345.06141	0.53	281(24), **330**(100), 331(14)	232(15), 271(22), 285(15), 287(13), 301(9), **315**(100), 316(12)	165(67), 175(23), 199(30), **243**(100), 259(33), 271(21), 287(50)
58	**Isorhamnetin**	7.69	$C_{16}H_{11}O_{7}^{-}$	315.05103	315.05084	0.59	**300**(100), 301(10)	**151**(100), 227(49), 228(25), 255(41), 271(85), 272(73), 283(32)	65(3), 83(5), **107**(100)
Anthocyanins and anthocyanidinds									
59	**Cyanidin 3,5-di-*O*-hexoside 1**	3.79	$C_{27}H_{31}O_{16}^{+}$	611.16066	611.16022	0.71	287(84), 288(11), **449**(100), 450(13)	**287**(100)	137(41), 175(29), 185(30), **213**(100), 231(55), 241(50), 287(92)
60	**Peonidin 3,5-di-*O*-hexoside**	4.06	$C_{28}H_{33}O_{16}^{+}$	625.17631	625.17494	2.20	301(69), 302(10), **463**(100), 464(17)	**301**(100)	258(4), **286**(100)
61	**Cyanidin 3-*O*-hexoside**	4.22	$C_{21}H_{21}O_{11}^{+}$	449.10784	449.10637	3.26	**287**(100), 288(12)	137(38), 175(30), 185(28), 213(87), 231(52), 241(45), **287**(100)	129(4), 141(21), 157(28), 167(9), 171(9), **185**(100), 213(7)
62	**Peonidin 3-*O*-hexoside**	4.67	$C_{22}H_{23}O_{11}^{+}$	463.12349	463.12236	2.43	**301**(100), 302(12)	258(3), **286**(100), 287(7)	202(9), 213(5), 229(5), 230(29), 257(17), **258**(100), 268(23)
63	**Cyanidin 3,5-di-*O*-hexoside 2**	5.05	$C_{27}H_{31}O_{16}^{+}$	611.16066	611.16009	0.93	303(24), 448(11), **449**(100)	**303**(100)	137(23), 153(22), 165(59), 229(85), 247(28), **257**(100), 285(52)
64	**Delphinidin 3-*O*-rhamnoside**	5.05	$C_{21}H_{21}O_{11}^{+}$	449.10784	449.10560	4.98	153(10), 287(12), **303**(100), 304(18), 418(60), 430(8), 431(5)	137(28), 153(25), 165(71), 229(94), 247(30), **257**(100), 285(50)	161(4), 201(8), **229**(100)
65	**Cyanidin 3-*O*-rhamnoside**	5.23	$C_{21}H_{21}O_{10}^{+}$	433.11292	433.11202	2.08	**287**(100), 288(8)	121(41), 153(71), **165**(100), 213(89), 231(32), 241(92), 258(49)	69(17), **109**(100), 137(89), 183(7)

**66**	**Petunidin 3-*O*-rhamnoside**	5.40	$C_{22}H_{23}O_{11}^{+}$	463.12349	463.12190	3.43	301(13), **317**(100), 318(22), 347(11), 395(9), 444(5), 445(10)	139(10), 257(10), 261(7), 274(10), 285(36), **302**(100), 303(16)	153(26), 246(8), 273(14), **274**(100), 275(5), 284(11), 285(16)
**67**	**Delphinidin**	5.50	$C_{15}H_{11}O_{7}^{+}$	303.04993	303.04919	2.45	137(28), 165(65), 229(93), 247(27), **257**(100), 285(75), 303(34)	161(3), 173(4), 201(10), 215(3), **229**(100)	145(9), 159(7), 161(22), 173(27), 183(12), 187(22), **201**(100)
**68**	**Cyanidin**	5.82	$C_{15}H_{11}O_{6}^{+}$	287.05502	287.05416	2.99	153(68), **165**(100), 213(85), 241(89), 258(46), 269(40), 287(47)	69(12), **109**(100), 137(97), 183(5)	
**69**	**Petunidin 3-*O*-hexoside**	6.02	$C_{22}H_{23}O_{12}^{+}$	479.11840	479.11644	4.10	**317**(100), 318(8)	139(9), 165(7), 257(10), 274(7), 285(38), **302**(100), 303(8)	153(25), 228(4), 246(9), 273(18), **274**(100), 284(10), 285(20)
Terpene derivatives									
**70**	**Desbenzoylpaeoniflorin**	1.82	$C_{16}H_{23}O_{10}^{-}$	375.12967	375.12916	1.36	195(6), 205(20), 215(7), 329(5), **341**(100), 343(11), 345(15)	101(18), 113(26), 119(26), 131(15), 143(26), 161(34), **179**(100)	**119**(100)
**71**	**Mudanpioside F**	3.45	$C_{17}H_{25}O_{10}^{-}$	389.14532	389.14491	1.06	161(38), 181(20), 325(24), 341(14), **343**(100), 344(11), 351(6)	113(24), 125(15), 143(29), 151(33), **161**(100), 163(59), 181(49)	
**72**	**Oxypaeoniflorin**	4.18	$C_{23}H_{27}O_{12}^{-}$	495.15080	495.15049	0.63	281(4), 299(4), 333(20), 334(4), **465**(100), 466(24), 477(9)	137(78), 165(33), 179(16), 209(12), 281(43), **299**(100), 327(31)	113(6), 137(54), 151(6), **179**(100), 209(19), 239(32)
**73**	**Paeonin B**	4.37	$C_{16}H_{21}O_{9}^{-}$	357.11911	357.11815	2.68	191(4), **195**(100), 196(11)	119(7), 123(14), 135(32), **136**(100), 151(74), 177(17), 180(4)	107(5), **121**(100)
**74**	**Mudanpioside E**	4.42	$C_{24}H_{29}O_{13}^{-}$	525.16137	525.16104	0.62	167(90), 363(41), 479(14), 491(13), **495**(100), 496(21), 507(19)	165(25), **167**(100), 209(23), 239(16), 311(33), 327(31), 329(50)	108(10), **123**(100), 152(27)
**75**	**Albiflorin + HCOOH**	5.35	$C_{24}H_{29}O_{13}^{-}$	525.16137	525.16099	0.72	**449**(100), 450(6), 478(5), 479(34)	165(25), 309(7), **327**(100)	113(5), 123(8), 149(4), **165**(100), 207(3), 309(20)
**76**	**Paeoniflorin**	6.83	$C_{23}H_{27}O_{11}^{-}$	479.15589	479.15605	-0.35	269(3), **271**(100), 432(8), 433(38)	**183**(100), 195(43), 211(15), 213(8), 225(7), 235(10), 253(77)	85(3), 97(19), **165**(100), 183(8)
**77**	**Benzoyl paeoniflorin + HCOOH 1**	7.55	$C_{31}H_{33}O_{14}^{-}$	629.18758	629.18745	0.21	535(3), **553**(100), 582(18), 583(71)	165(21), 245(4), 413(6), **431**(100)	**165**(100), 179(3), 217(3), 265(5), 291(3), 309(11), 413(21)
**78**	**Paeoniflorigenone**	7.67	$C_{17}H_{19}O_{6}^{+}$	319.11762	319.11683	2.45	179(57), **197**(100), 257(7), 285(24), 301(7), 302(69), 303(11)	123(3), 137(5), 151(4), 161(6), 167(11), **179**(100)	**133**(100), 151(17), 161(57)
**79**	**Benzoyl paeoniflorin + HCOOH 2**	8.27	$C_{31}H_{33}O_{14}^{-}$	629.18758	629.18792	-0.53	**553**(100), 554(17), 582(17), 583(100), 584(8)	165(14), 413(6), **431**(100), 535(3)	123(5), 150(4), **165**(100), 227(5), 249(4), 309(11), 413(7)
**80**	**Benzoyl paeoniflorin + HCOOH 3**	9.36	$C_{31}H_{33}O_{14}^{-}$	629.18758	629.18760	-0.03	445(3), 582(12), **583**(100), 584(27)	177(22), 195(26), 299(19), 387(8), 461(9), **461**(100), 565(5)	133(36), 151(22), 165(9), **177**(100), 195(58), 545(31)
Other compounds									
**81**	**Shikimic acid**	0.78	$C_{7}H_{9}O_{5}^{-}$	173.04555	173.04548	0.38	93(58), **111**(100), 127(29), 129(43), 137(19), 143(22), 155(58)	57(15), 67(11), 81(5), 83(57), **93**(100)	
**82**	**Citric acid**	1.01	$C_{7}H_{11}O_{6}^{-}$	191.05611	191.05597	0.74	85(63), 93(34), **111**(100), 127(47), 145(13), 171(18), 173(48)	67(79), 81(10), 83(32), **93**(100), 96(21)	
**83**	**Pinoresinol hexoside**	5.80	$C_{26}H_{31}O_{11}^{-}$	519.18719	519.18716	0.04	**357**(100), 358(13), 469(4), 485(4)	136(40), **151**(100), 311(10), 327(7), 342(11)	**136**(100)
