# Supplementary material for: Chemical Profile and Skin-Beneficial Activities of the Petal Extracts of Paeonia tenuifolia L. from Serbia
Source: Pharmaceuticals (Basel). 2022 Dec 11;15(12):1537. doi: 10.3390/ph15121537 (PMC9787298; doi:10.3390/ph15121537)
Supplement: Supplementary file 1 [file pharmaceuticals-15-01537-s001.zip › Table S2.pdf]

Table S2 Quantification of active compounds in the extracts of the petals of *P. tenuifolia* L.

Origin of plant material	Extraction medium	Extraction method	***p*-Coumaric acid**, mg/L	**Ellagic acid**, mg/L	**Quercetin**, mg/L	**Isorhamnetin**, mg/L
Gulenovci	H_2O	Maceration	0.253±0.008^{l*}	166.33±14.62^{a}	0.386±0.006^{i}	0.52±0.02^{j}
		UAE	0.70±0.02^{g}	143.10±12.58^{ab}	0.312±0.004^{j}	0.43±0.02^{k}
		MAE	NF	124.00±10.90^{bc}	0.159±0.002^{o}	0.26±0.01^{m}
	MeOH	Maceration	0.456±0.014^{h}	80.03±7.03^{e}	0.188±0.001^{n}	0.45±0.02^{k}
		UAE	0.290±0.009^{k}	84.87±7.46^{e}	0.595±0.009^{f}	1.87±0.08^{f}
		MAE	1.56±0.05^{e}	47.24±4.15^{g}	0.411±0.006^{h}	1.54±0.06^{g}
Pančevo	H_2O	Maceration	15.04±0.45^{a}	72.63±6.38^{ef}	0.209±0.003^{m}	5.23±0.21^{b}
		UAE	2.30±0.07^{d}	35.49±3.12^{h}	1.34±0.02^{c}	7.22±0.29^{a}
		MAE	1.52±0.05^{e}	102.41±9.00^{d}	0.947±0.014^{d}	3.19±0.13^{d}
	MeOH	Maceration	4.57±0.14^{b}	81.83±7.19^{e}	0.502±0.007^{g}	3.65±0.15^{c}
		UAE	4.28±0.13^{b}	85.86±7.55^{e}	0.619±0.009^{e}	3.54±0.14^{c}
		MAE	2.70±0.08^{c}	75.04±6.60^{ef}	0.321±0.005^{j}	2.37±0.10^{e}
Deliblato sands	H_2O	Maceration	0.79±0.02^{f}	106.49±9.36^{cd}	0.244±0.003^{l}	0.64±0.03^{i}
		UAE	0.257±0.008^{l}	103.81±9.13^{d}	0.265±0.004^{k}	0.39±0.02^{l}
		MAE	NF	50.35±4.43^{g}	0.585±0.008^{f}	0.64±0.03^{i}
	MeOH	Maceration	NF	125.26±11.01^{bc}	3.15±0.05^{a}	6.77±0.28^{a}
		UAE	0.386±0.011^{j}	63.49±5.58^{f}	1.85±0.03^{b}	3.61±0.15^{c}
		MAE	0.415±0.012^{i}	30.67±2.30^{h}	0.581±0.008^{f}	1.078±0.04^{h}

[*]values with different letters in each column showed significant differences ($p<0.05$; n=3; analysis of variance, Duncan's post-hoc test)
