# Supplementary figures and images for: Chemical Profile and Skin-Beneficial Activities of the Petal Extracts of Paeonia tenuifolia L. from Serbia
Source: Pharmaceuticals (Basel). 2022 Dec 11;15(12):1537. doi: 10.3390/ph15121537 (PMC9787298; doi:10.3390/ph15121537)

**Figure S1** Trolox calibration curve for DPPH• radical scavenging activity.

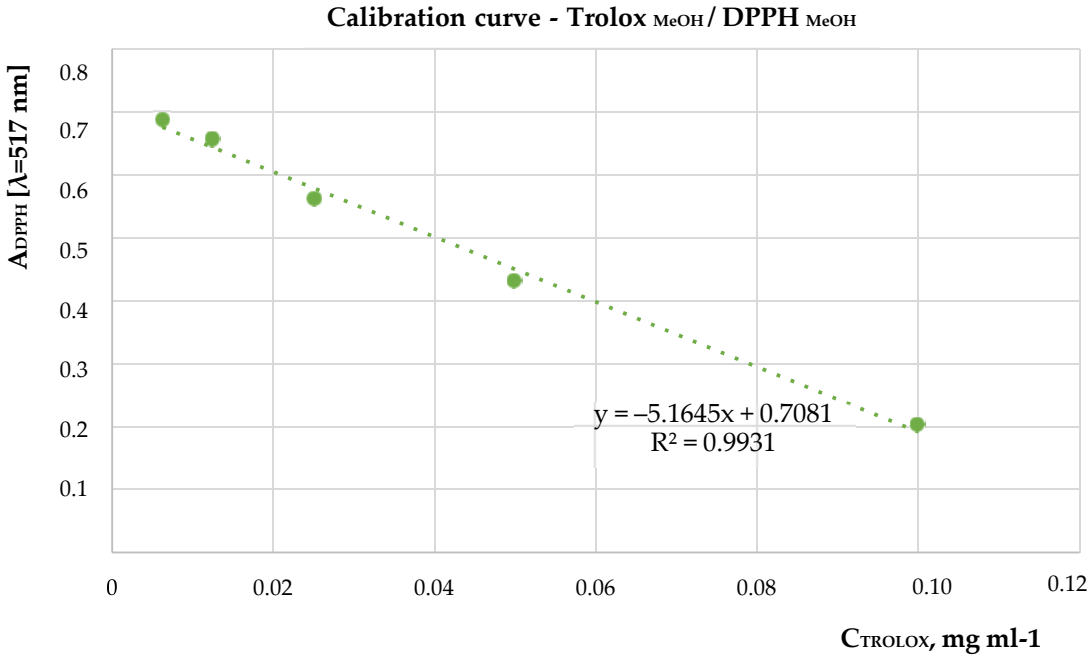

Supplement: Supplementary file 1 [file pharmaceuticals-15-01537-s001.zip › Figure S1.pdf]
